# The perceived deservingness of COVID-19 healthcare in the Netherlands: a conjoint experiment on priority access to intensive care and vaccination

**DOI:** 10.1186/s12889-021-10488-3

**Published:** 2021-03-05

**Authors:** Tim Reeskens, Femke Roosma, Evelien Wanders

**Affiliations:** School of Social and Behavioral Sciences, Tilburg University, PO Box 90153, 5000 LE Tilburg, The Netherlands

**Keywords:** Conjoint experiment, COVID-19, Deservingness theory, ICU, LISS panel, Netherlands, Vaccination

## Abstract

**Background:**

Amidst the COVID-19 pandemic, governments, health experts, and ethicists have proposed guidelines about ICU triage and priority access to a vaccine. To increase political legitimacy and accountability, public support is important. This study examines what criteria beyond medical need are deemed important to be perceived of priority COVID-19 healthcare access.

**Method:**

Two conjoint experiments about priority over ICU treatment and early COVID-19 vaccination were implemented in a probability-based sample of 1461 respondents representative of the Netherlands. Respondents were asked who should receive treatment out of two fictitious healthcare claimants that differed in in age, weight, complying with corona policy measures, and occupation, all randomly assigned. Average marginal coefficient effects are estimated to assess the relative importance of the attributes; attributes were interacted with relevant respondent characteristics to find whether consensus exists in this relative ranking.

**Results:**

The Dutch penalize those not complying with coronavirus policy measures, and the obese, but prioritize those employed in ‘crucial’ sectors. For these conditions, there is consensus among the population. For age, young people are prioritized for ICU treatment, while the middle-aged are given priority over a vaccine, with younger respondents favoring healthcare for elderly claimants, while older respondents favor support for young cohorts.

**Conclusion:**

People who have no control over their social risk and are able to reciprocate to society are considered as more deserving of priority of COVID-19 healthcare. Our findings provide fair support for the implemented ethical guidelines about ICU-treatment and COVID-19 vaccines.

**Supplementary Information:**

The online version contains supplementary material available at 10.1186/s12889-021-10488-3.

## Introduction

From the onset of the COVID-19 pandemic, it appeared that not every life counts the same, with some (like the elderly and patients with underlying health conditions) more likely to develop adverse medical consequences from SARS-CoV-2 [1, 2]. To cope with an unprecedented influx of COVID-19 patients, ethical guidance was released in case a systems of ICU triage was required [3, 4]. Similar ethical debates took place about the allocation of a scarce first release of COVID-19 vaccines, as early production capacity is limited [5]. These ethical guidelines often converge in the utilitarian principle of maximizing benefits [6] to “save the greatest number of lives or preserve the largest amount of life-years among treated patients” [7].

Public opinion has been largely left outside of these ethical debates, even though studies suggest that public policy requires support by the public to work efficient and effectively [8, 9], to ensure democratic accountability [10, 11], and to build public confidence in political and social institutions in the face of a pandemic [12, 13]. In this contribution, we ask public opinion, representative of the Dutch population, who is perceived as more deserving of priority (a) over ICU care, and (b) in COVID-19 vaccination. Doing so, we extend a growing body of publications focused on ICU triage dilemmas [7, 14] by including preferences towards the allocation of scarce vaccines, and by zooming in on the Netherlands.

The few existing studies on COVID-19 ICU triage, conducted Britain [14] and the US [7], show that the maximizing benefits principle also prevails among public opinion. Our study takes a different theoretical approach by considering healthcare applicants’ attributes that largely go beyond medical frailty. Deservingness theory [15, 16] has identified five criteria to determine why some are perceived as more deserving of support, namely *control* over social risks, *attitude* towards received support, *reciprocity* to its contributors, the extent to which a person’s *identity* is close to the contributor, and his/her *need* for social support. Social changes towards increased individualization with a corresponding “privatization of social risks” [17], and a welfare state that shifted from protecting people against abuses from the market towards integrating them into the market [18], underscore a growing importance of the *control* and *reciprocity* criteria when it comes to perceived welfare deservingness [19].

However, applied to the perceived deservingness of healthcare, the criterion of *need* plays a dominant role [20, 21]. Because the healthcare system is essentially a “need-driven system” [21], public perceptions of deservingness is foremost determined by the level of *medical need* of the target group. The COVID-19 pandemic alters this logic. Confronted with a scarcity of ICU treatments and vaccines, priorities have to be made among persons who are in equal need of care. Precisely because recent studies identified that public opinion endorses triage based on the ethical principle that of maximizing benefits [7, 14], the opportunity exists to examine the relevance of perceived deservingness criteria that go beyond medical need.

The criteria we evaluate in our study have frequently been discussed in ethical considerations and public debates, but also align well with deservingness theory, namely a healthcare claimant’s age, weight, occupation and compliance with corona policy regulations. Age aligns to *reciprocity*, and it matters because a younger person is able to give back more to society in the future, making them perceived as more deserving. Weight reflects *control*, as it signals individual lifestyles (making them perceived as less deserving). Obeying the coronavirus policy measures mixes *control* (higher likelihood to have caught the corona virus because of noncompliance) with an unfavourable *attitude* toward the collective effort to get the pandemic under control. In both cases the person is considered less deserving. The fourth and final attribute is occupation. Here were capture *reciprocity*, as we assume that being employed in ‘crucial’ occupations (healthcare and education) makes them perceived as more deserving.

## Methodology

### Data source

Two unique survey experiments were integrated in the LISS Panel [22], which relies on a probability sample representative of the Dutch population. The study is part of a continuous monitoring of values change and stability amidst the COVID-19 pandemic [23]. Of the 1601 panel members that were invited to participate, 1461 respondents (91.3%) completed the questionnaire. The data collection took place from 5 to 27 October 2020, which was at the onset of the second wave of the COVID-19 pandemic in the Netherlands. To correct for the Dutch population’s distribution with regard to sex, age, education, and region, post-stratification weights are applied. The dataset analysed during the current study are available from the corresponding author on reasonable request.

### Two conjoint experiments

A choice-based conjoint design was implemented, which has some relevant applications in the study of preferences in social science research [24]. Following a short introduction, respondents were offered a table that showed two distinct profiles of, in random order to exclude carryover effects, healthcare claimants requesting ICU treatment and vaccination. A forced-choice experiment has the advantage that respondents need to make trade-offs [24], excluding the possibility that egalitarian values influence their preferences.

The conjoint experiment about priority access to the ICU was preceded with the following introduction:

Early in the coronavirus crisis, the government and experts were concerned that the intensive care unit would be overwhelmed with COVID-19 patients. Although it has not yet happened that the intensive care unit could no longer cope with the inflow of COVID-19 patients, considerations are made which patients should be given priority over ICU beds. Below you can find two descriptions of COVID-19 patients whose doctors estimate the probability of survival equally. Which of the two do you think should be given priority?

The conjoint experiment about priority for a COVID-19 vaccination was preceded with this introduction:

Currently, the pharmaceutical industry is working hard developing a vaccine against COVID-19. Since the production of the vaccine on such a large scale takes time, it most likely will not be readily available to everyone. Below you will find two descriptions of persons who would like to be vaccinated. If the vaccine is not available to everyone, who do you think should be given priority in vaccination?”

### Operationalization of attributes

For age, we differentiate between a patient of 27, 52 and 77 years old. For weight, we contrasted people with a healthy (BMI = 22) and unhealthy weight (BMI = 31). For obeying the coronavirus policy measures, the ideal operationalization of wearing a facial mask was impossible because during the timing of the fieldwork, there was no nationwide mask mandate; alternatively we looked at following travel recommendations, distinguishing between a patient that went on holiday to Barcelona in spite of a negative travel advice, and a patient who cancelled this holiday after negative travel recommendations. Lastly, for professional situation, we distinguish between a nurse, a teacher, an administrative assistant, and an unemployed healthcare claimant. The professional situation attribute explicitly mentions “before retirement” in case the oldest attribute (77 years old) was presented, as by law it is prohibited to practice salaried work after the age of 67.

Respondents were offered the descriptions of two randomized people (per conjoint experiment) as presented in Table 1. As is necessary in such experiments for valid inference, attributes were randomly assigned to the respondents, leading to the possibility of 48 different combinations (3*2*2*4). In case of an identical description, it was decided that the respondent would be offered a different occupation.

**Table 1:** Experimental Design of the Two Conjoint Experiments

Characteristics	Person A:	Person B
Age	• 27 years old • 52 years old • 77 years old	• 27 years old • 52 years old • 77 years old
Weight	• Healthy weight (BMI = 22) • Unhealthy weight (BMI = 31)	• Healthy weight (BMI = 22) • Unhealthy weight (BMI = 31)
Compliance with COVID-19 measures	• Went on holiday to Barcelona in spite of a negative travel advice • Cancelled a holiday to Barcelona when a negative travel advice was given	• Went on holiday to Barcelona in spite of a negative travel advice • Cancelled a holiday to Barcelona when a negative travel advice was given
Occupation (before retirement)	• Nurse • Teacher • Administrative assistant • Unemployed	• Nurse • Teacher • Administrative assistant • Unemployed

### Statistical analysis

We present the *average marginal component effects* (AMCEs) [25], which “represents the average difference in the probability of being preferred ( …) when comparing two different attribute values ( …) where the average is taken over all possible combinations of the other ( …) attributes” [24]. The output tables can be found in Online Additional file 1 (Section 2). We present the AMCE plots; for each attribute value presented in the graphs, the dots indicate the point estimates, and the lines illustrate the 95% confidence intervals for the AMCE. The top row indicates the reference category per criteria. The analyses are conducted in RStudio, using the *cjoint* package to estimate the AMCEs. Syntax can be retrieved from Online Additional file 1 (Section 3). All methods were carried out in accordance with relevant guidelines and regulations.

### Ethical disclosure

The funding source of this study had no role in the design, data analysis, interpretation, or writing this manuscript. Ahead of the study, ethical approval was asked and granted by the Ethical Review Board of the Tilburg School of Social and Behavioral Sciences. Informed consent was obtained from all panel participants. Nevertheless, both experiments confronted participants with tough moral choices. At the end of the survey, 49 respondents voiced concerns about the choice-based conjoint experiment, with for instance one respondent stating “I found it very difficult to make a choice: actually I’d rather not do this. I hope never to be in a position to actually make this choice.”

## Findings

### Effect of attributes on the perceived deservingness of COVID-19 healthcare

**Fig. 1:**
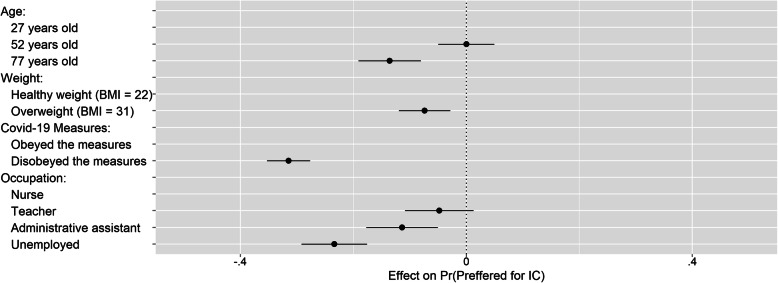
Effects of Patient Attributes on Probability of Being Given Priority over ICU Treatment. Note: This plot shows estimates of the effects of the randomly assigned patient attribute values on being given priority over ICU treatment. Bars represent 95% confidence intervals

**Fig. 2:**
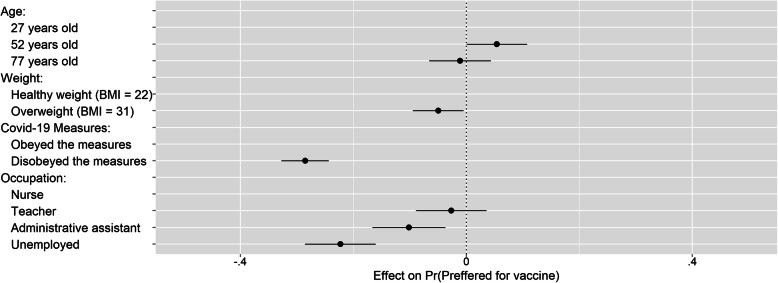
Effects of Vaccine Claimant on Probability of Being Given Priority over a COVID-19 Vaccine. Note: This plot shows estimates of the effects of the randomly assigned vaccine claimant attribute values on being given priority over a COVID-19 vaccine. Bars represent 95% confidence intervals

The analysis of the AMCEs (see Figs. 1 and 2) is, with some noteworthy exceptions, surprisingly similar for who should receive priority over ICU treatment, and over a COVID-19 vaccine. Most relevant is compliance with the coronavirus policy measures (proxy for both *control* over and *attitude* towards own social risk), with those not obeying to the measures receiving on average 31.5 percentage points (SE = 0.02) less support compared with those obeying the rules in case of ICU access. Also in terms of priority access over vaccination, non-compliant claimants receive on average 28.5 percentage points (SE = 0.02) less priority by the Dutch population.

Second most relevant is claimant’s occupation, which reflects *reciprocity*. Compared to being a nurse, an unemployed patient is 23.4% (SE = 0.03) less likely to be given priority over an ICU bed, while this is 11.48% for an administrative assistant (SE = 0.03). Similar coefficients are found for priority over the first COVID-19 vaccines, with the unemployed claimant or administrative assistant given respectively 22.3 percentage points (SE = 0.03) and 10.1 percentage points (SE = 0.03) less priority compared to a nurse. Interestingly, for both ICU access and COVID-19 vaccinations, no significant effects are found for the teacher attribute, possibly due to the fact that teachers and nurses are deemed ‘critical’ jobs during the pandemic.

The third attribute that shows consistent patterns over the two experiments is the claimant’s weight, which theoretically combines *control*. For being perceived as deserving of priority access to the ICU, a penalty for obesity exists (− 7.4%, SE = 0.02). In case of priority vaccine access, the overweight has a five percentage points (SE = 0.02) lower chance of being prioritized compared to a claimant with a normal weight (BMI of 22).

In final, age – a proxy for *reciprocity* – shows diverging results for the perceived priority access to ICU and a vaccine. Compared to a person of 27 years old, elderly are 13.6 percentage points (SE = 0.03) less perceived to be prioritized at ICU care. For the age of 52, there is no significant difference from baseline. The results of the vaccine experiment are different: a person aged 52 is 5.4 percentage points (SE = 0.03) more likely to be prioritized for a COVID-19 vaccine compared to someone of 27. There are no significant differences in the perceived COVID-19 deservingness of the youngest and oldest claimant. Results thereby confirm the importance of the *reciprocity* criterion.

### Interactions with respondent characteristics

**Fig. 3:**
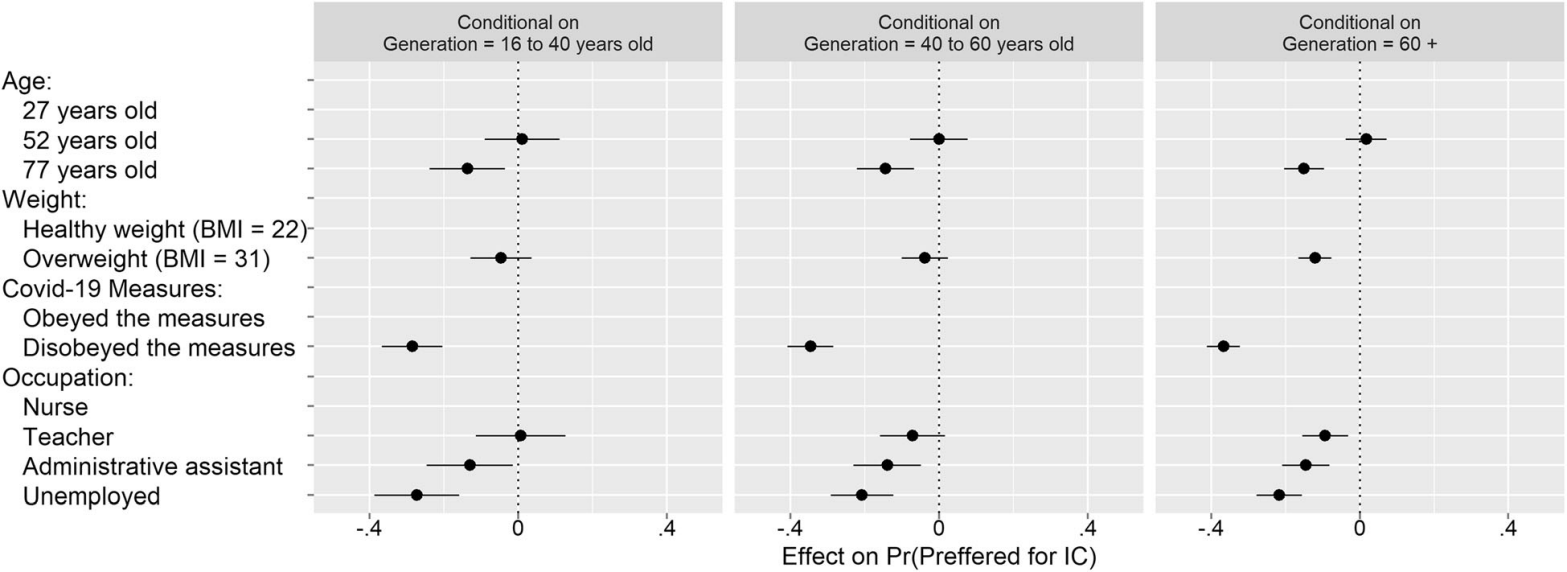
Effects of Patient Attributes on Probability of Being Given Priority over ICU Treatment by Age Cohort. Note: These plots show estimates of the effects of the randomly assigned patient attribute values on being given priority over ICU treatment for respondents aged 16–40, 40–60, and 60+. Bars represent 95% confidence intervals

**Fig. 4:**
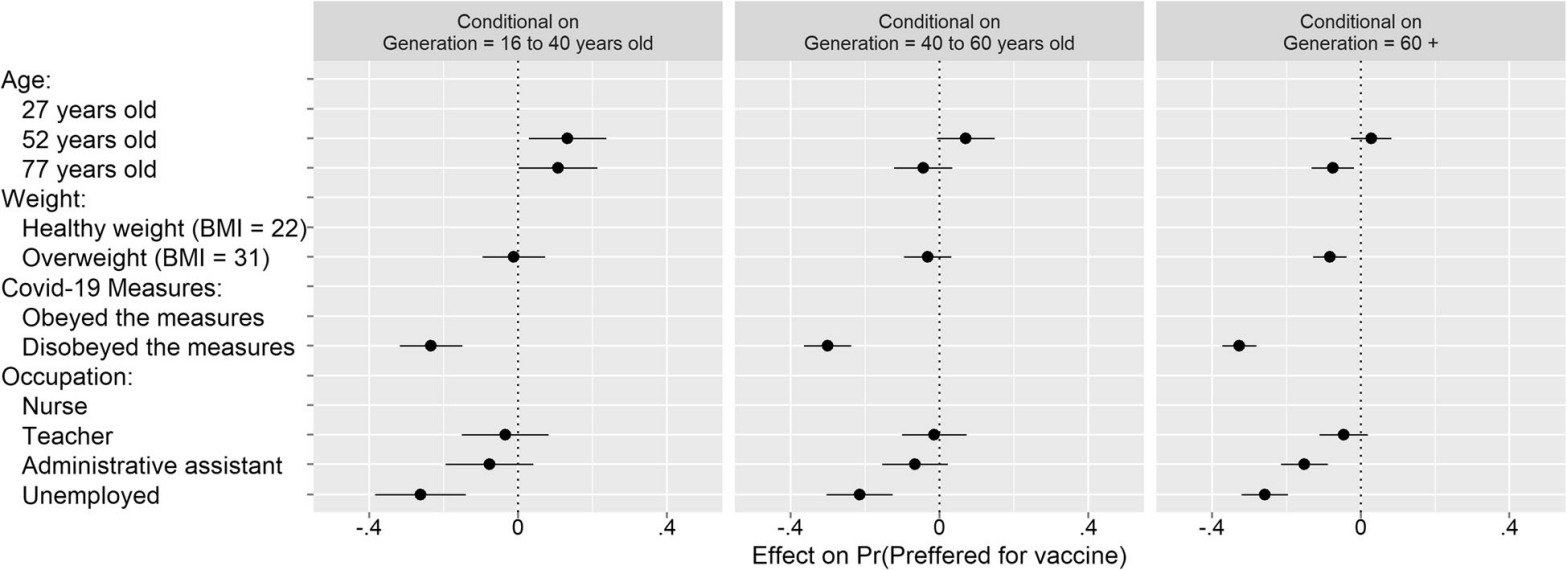
Effects of Vaccine Claimant on Probability of Being Given Priority over a COVID-19 Vaccine by Age Cohort. Note: These plots show estimates of the effects of the randomly assigned vaccine claimant attribute values on being given priority over a COVID-19 vaccine for respondents aged 16–40, 40–60, and 60+. Bars represent 95% confidence intervals

To further leverage our findings, we assessed the influence of respondent characteristics in their priorities over who should receive ICU access and an early COVID-19 vaccine (see Figs. 3 and 4). Empirical research suggests the importance of two paradigms, namely self-interest and ideology [26]. Self-interest implies punishing those with criteria different from the respondent, as they might create a competitive advantage in obtaining the scarce care [26]. For ideology, the expectation is that respondents at the left are more egalitarian, thereby showing less outspoken priorities compared to respondents who identify with the political right [27]. All estimated interactions can be retrieved from the Online Additional file 1 (Section 1).

While self-interest predicts that respondents would choose their own generation, our data shows opposing patterns. In case of ICU access all respondents preferred the youngest over the oldest. In case of the perceived priority over a COVID-19 vaccine, the youngest cohort in our sample (16–40 years) gives priority to the people older than themselves: a 77-year-old person the chance of being perceived as deserving of an early vaccine is 20.7% (SE = 0.05) higher compared to the baseline (claimant of 27 years old). On the contrary, the oldest cohort in our sample, i.e. respondents over 60 years, prioritized the care seeker with the age of 27 years, as those 77-years old are perceived 7.5 percentage points (SE = 0.03) less deserving of a vaccine. The group respondents between forty and sixty years of age show no differences in prioritization.

In our questionnaire, we also asked respondents to assess their health, which also serve as a test for self-interest. Respondents considering themselves healthy penalized someone who is overweight; for ICU access, an overweight patient is 9.6 percentage points (SE = 0.02) less preferred, for the vaccine, this is reduced to 6.6 percentage points (SE = 0.02). By contrast, those who consider themselves unhealthy do not make a choice based on the weight characteristic.

For self-interest, we also asked whether respondents comply with the social distance measures themselves, examining if it is related to giving more priority to those following government advice. Here, we see no strong indications of self-interest. Although people who indicate to follow the social distance measures perceive those who are compliant with the rules as more deserving of ICU access and an early COVID-19 vaccine, the differences are rather small.

The paradigm of political ideology, then, is estimated by interacting respondent’s self-assessed left-right placement with the presented attributes. In terms of ICU-access, there is limited evidence that the left hold vastly different priorities compared to the right (although differentiation tends to be larger among right-wing respondents), with two noteworthy exceptions. Left-wing respondents do not strongly distinguish between occupations, with only the unemployed receiving less support (− 18.8%, SE = 0.04) compared with nurses. Right-wing respondents make clear distinctions, with the nurse claimant most deserving of an ICU-bed, followed by the teacher (− 9.1%, SE = 0.03), the administrative assistant (− 17.5%, SE = 0.04) and the unemployed (− 26.3%, SE = 0.03). Also obeying the coronavirus policy measures is relevant, as patients who do not obey the social distance measure are 36.8% (SE = 0.02) less likely to receive priority from a right-wing respondents; among left-wing respondents, this effect is slightly smaller at 32.2% (SE = 0.03).

In case of priority access over a COVID-19 vaccine, left-wing and right-wing respondents show largely similar response patterns, with a few interesting exceptions. Left-wing respondents are more likely to penalize claimants disobeying the coronavirus measures (which is different from the ICU experiment), and penalize the obese. Compared to left-wing voters (− 32.5%, SE = 0.03), right-wing voters are about 5 percentage points less strict with disobedient people (− 27.8%, SE = 0.03). Left-wing voters show less support for overweight people (− 5.3%, SE = 0.03).

## Discussion

While governments have consulted and are relying on health experts and medical ethicists in political decisions about ICU triage and about which groups should be prioritized over others in the early release of a COVID-19 vaccine, insights from public opinion have largely focused on broader ethical principles [7, 14], providing a window of opportunity to empirically verify allocation criteria derived from deservingness theory [15]. While studies on deservingness of healthcare underscore the importance of *medical need* to be considered deserving, a pandemic is unique because priorities need to be made between people equally in need of care. To our knowledge, this is the first study on the perceived deservingness of COVID-19 healthcare that distinguishes between access to ICU treatment and an early COVID-19 vaccine.

Among a sample representative of the Dutch population, a general consensus exists that individual *control*, *reciprocity* and a favourable *attitude* massively influence the perceived deservingness of COVID-19 healthcare. Noncompliance with the coronavirus measures (i.e. *control* over contracting the coronavirus and a negative *attitude* towards the collective effort of containing the virus) trumps solidarity. While one might reverse the causality, i.e. people who don’t want to have priority access to the vaccine are those who are also less likely to obey the rules, reversed causality is unlikely because this logic clearly does not apply for priority access to the ICU. Policy-wise, this finding is also important: it underscores that the Dutch favour penalizing those not abiding to the rules. Also, employment in ‘crucial’ sectors, like health care workers and lesser so teachers, which are able to reciprocate to vulnerable people, are perceived more deserving.

The relevance of the criteria of *control* and *reciprocity* cannot be isolated from the ethical principle of maximizing benefits. On the one hand, noncompliance with the COVID-19 measures jeopardizes public health; in line, the population prefers to prioritize scarce healthcare resources to those that contribute to public health by complying with government measures instead of rewarding behaviour that puts public health at risk. On the other hand, employment in ‘crucial’ sectors follows a similar analogy: putting nurses in front of the line of scarce ICU care and vaccines assures that healthcare system does not collapse in case healthcare workers are exposed to the virus. Our study thereby confirms the dominance of the maximizing benefits-principle in the allocation of scarce COVID-19 healthcare.

In addition, age underscores the important role of *reciprocity*, even though the attribute relevance of age adds complexity to our initial argument. For curative healthcare, i.e. ICU treatment which poses the situation that both patients have an equal medical need and chance of survival, less priority is given to the oldest groups. However, for preventive care of vaccination, *reciprocity* triumphs over medical need, as the general public wants to grant the active population priority access to a vaccine, leaving the more vulnerable elderly behind. Age also appears as a reason for altruism: the younger are more likely to support the elderly in terms of an early COVID-19 vaccine, and vice versa. A general fear over the vaccine as alternative explanation is less supported, because the overall consensus present in the rankings.

Additionally interesting is the attribute of weight. People suffering from obesity are put at the end of the line in the access to ICU care (in case of equal chances on survival), showing the importance of *control* (over social risks); people with a presumed unhealthy lifestyle are punished when they make an equal claim to access to healthcare. This is even more remarkable in the case of perceived access over a vaccine. As far as we know, the COVID-19 vaccine does not discriminate for weight and equally protects people of different weight against infection. Persons with a higher BMI therefore could be seen rather as more deserving of preventive care because they are at risk to suffer more severely from COVID-19. Not granting people with higher weight priority access, seems to suggest that not deservingness criteria play a role but rather negative stereotyping and possibly discrimination of people with obesity.

Although some respondents articulated unease, this research underscores the relevance of using conjoint experiments in the study of public opinion on difficult ethical considerations, such as priority access over COVID-19 health care. By asking respondents to choose between two healthcare claimants, more general egalitarian attitudes where all claimants would be deemed deserving of healthcare do not influence individual priorities. This is most obvious in the widespread consensus that exists among the Dutch population. The rank order of important deservingness criteria is shared among large sections of the population that in traditional survey research reports rather diverging views in terms of solidarity. Also our two most surprising findings, namely that the elderly are not perceived as most deserving of COVID-19 healthcare, and that the obese are disfavoured, would be more difficult to tease out in more traditional observational studies.

From a political perspective, the results give also support for the measures that are being undertaken or overthought. Employment in ‘crucial’ sectors, like healthcare, is being considered as a guideline for priority access to healthcare amidst the COVID-19 pandemic, following the utilitarian principle of benefits maximizing, and is also endorsed by public opinion. The most important discrepancy lies in the ‘subjective’ factor of compliance with the coronavirus measures. While examples exist of mediatized very first vaccinations given to elderly compliant with the COVID-19-rules [28], at a more general level it is basically impossible to implement this criterion, as there is no register that documents how compliant people have been with coronavirus measures. Our findings nevertheless show that public opinions wants to penalize people that jeopardize public health. More open to further reflection are age and BMI, which show patterns opposing to ethical recommendations. In sum, our study overall provides further legitimacy to political and social institutions responsible for ICU triage and the allocation of scarce vaccines.

## Supplementary Information

**Additional file 1.**

## Data Availability

The datasets analysed during the current study are available from the corresponding author on reasonable request.
