# Supplementary material for: The perceived deservingness of COVID-19 healthcare in the Netherlands: a conjoint experiment on priority access to intensive care and vaccination
Source: BMC Public Health. 2021 Mar 5;21:447. doi: 10.1186/s12889-021-10488-3 (PMC7934976; doi:10.1186/s12889-021-10488-3)
Supplement: Supplementary file 1 — Additional file 1. [file 12889_2021_10488_MOESM1_ESM.docx]

**SUPPLEMENTARY FILE**

**The Perceived Deservingness of COVID-19 Healthcare in the Netherlands:**

**A Conjoint Experiment on Priority Access to Intensive Care and Vaccination**

**Tim Reeskens, PhD***

Associate Professor of Sociology

Tilburg University
School of Social and Behavioral Sciences
PO Box 90153
5000 LE Tilburg
The Netherlands

[t.reeskens@tilburguniversity.edu](mailto:t.reeskens@tilburguniversity.edu)
0031 13 466 2119

**Femke Roosma, PhD**

Assistant Professor of Sociology

Tilburg University
School of Social and Behavioral Sciences
PO Box 90153
5000 LE Tilburg
The Netherlands

**Evelien Wanders, BSc**

Student Master ‘Health, Wellbeing and Society’

Tilburg University
School of Social and Behavioral Sciences
PO Box 90153
5000 LE Tilburg
The Netherlands

* Corresponding author

**Sections:**

1. **Relevant Interaction Graphs**
2. **Output Average Marginal Coefficient Effects**
3. **Syntax**

**SECTION 1: INTERACTION PLOTS**

1. **Priority Access over ICU Treatment**


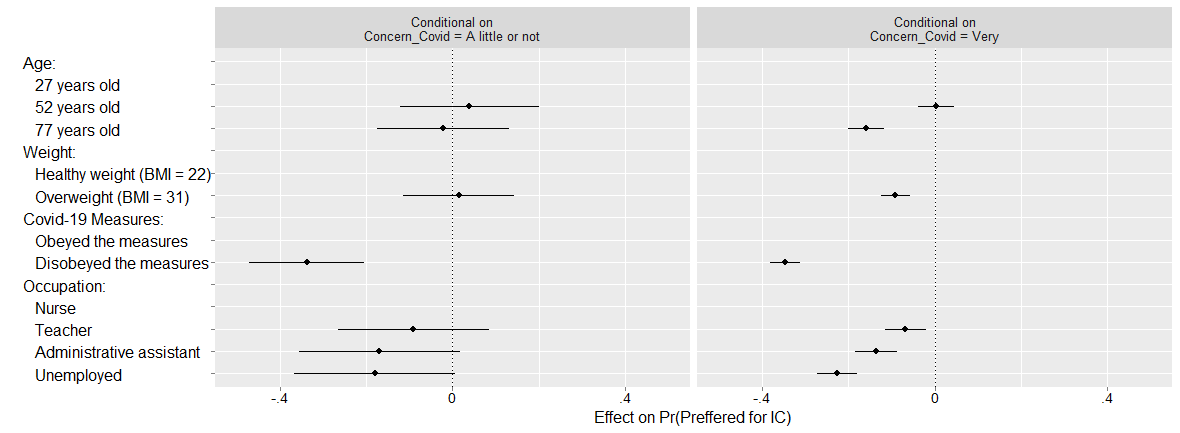

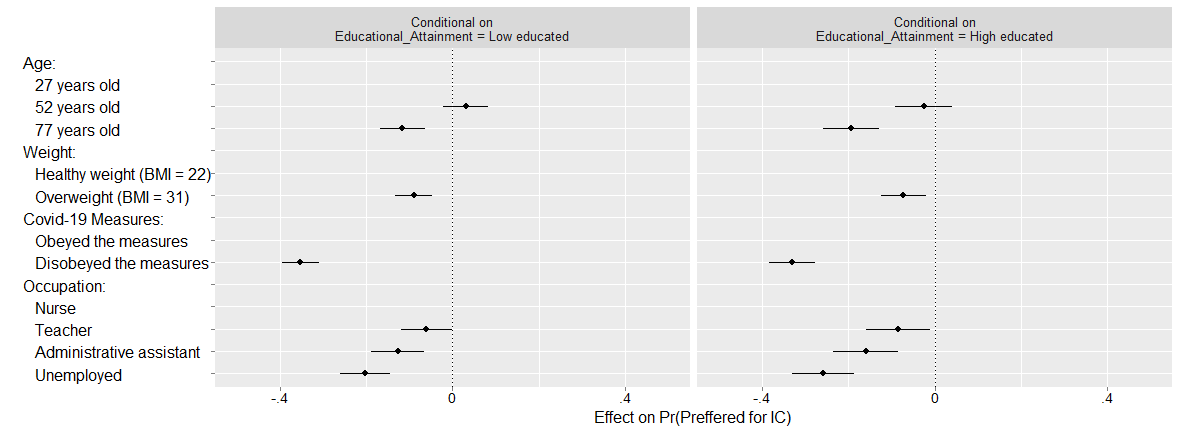

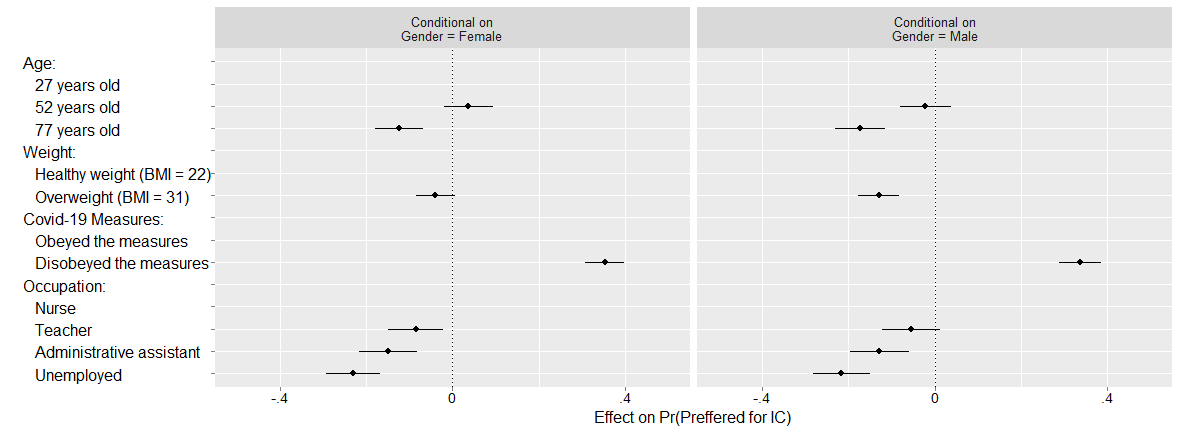

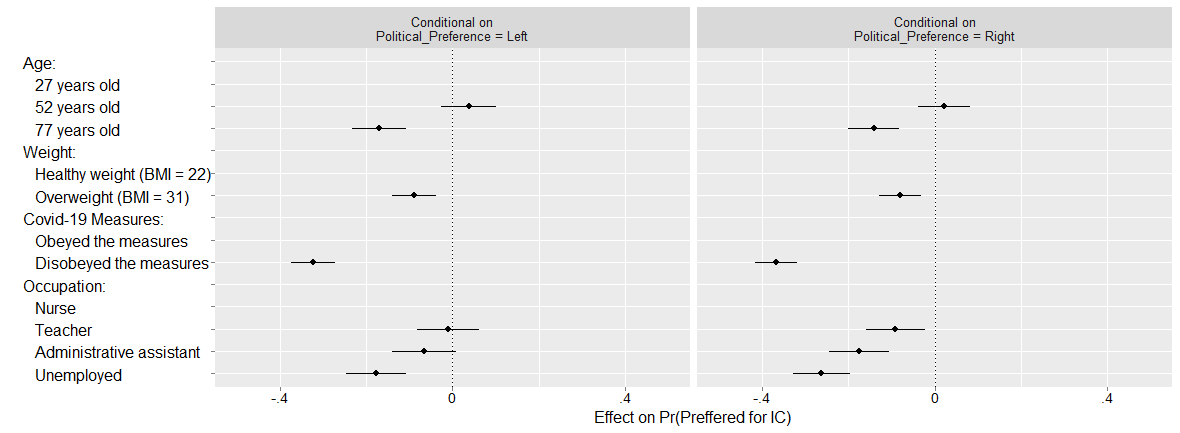

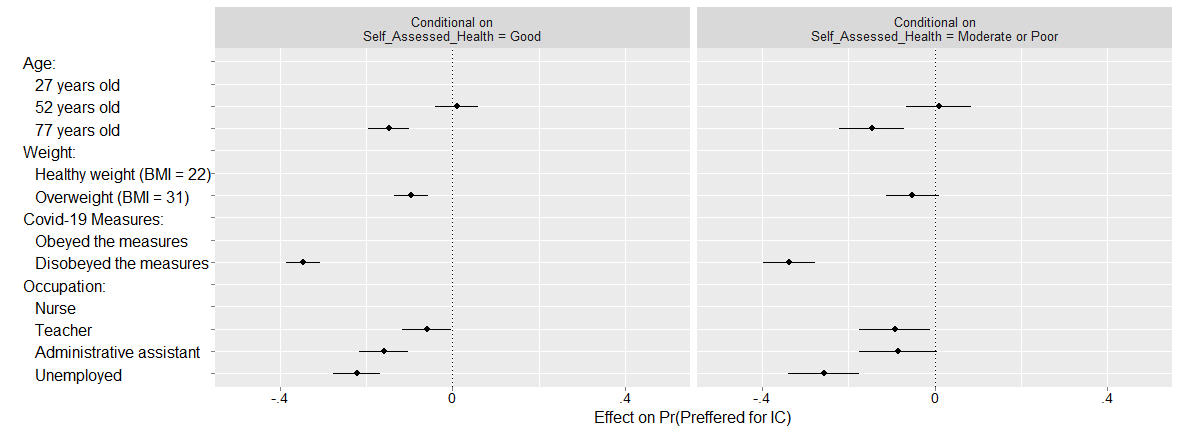

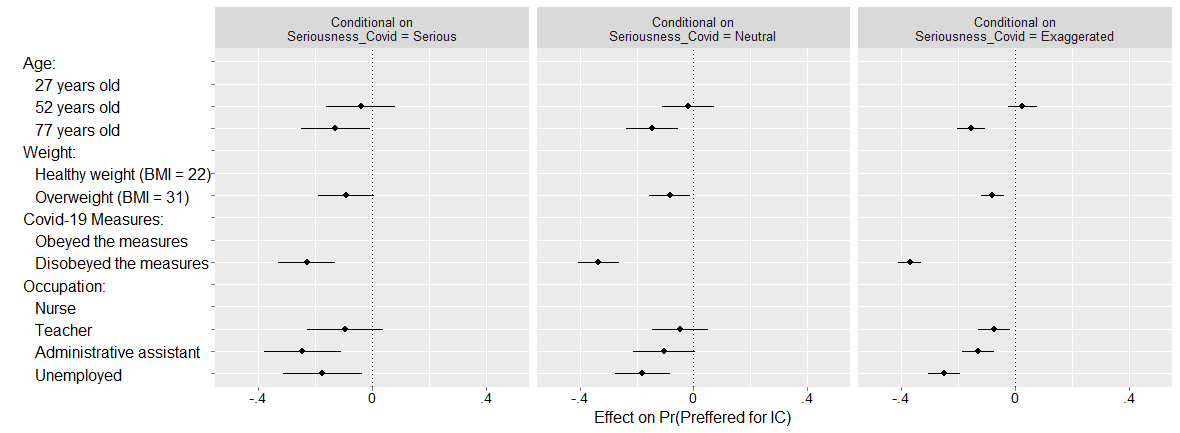

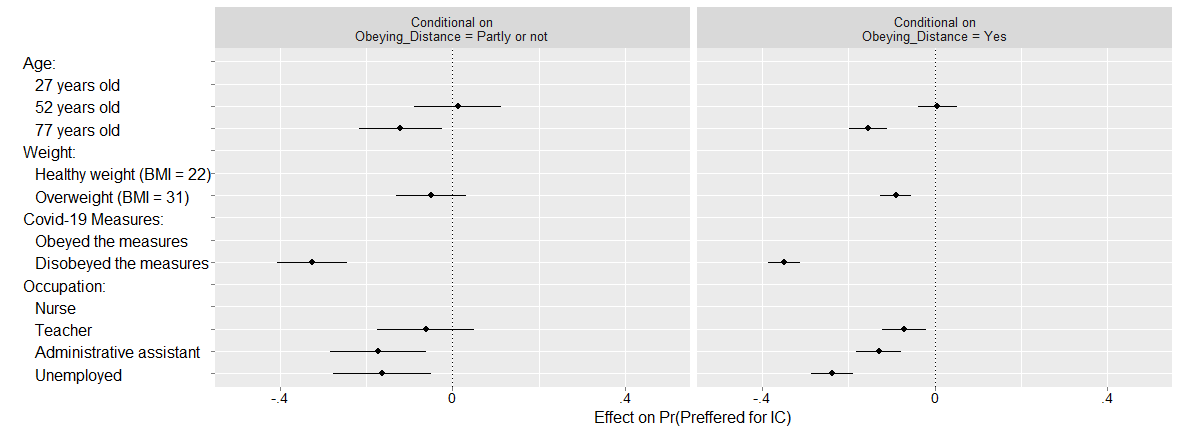

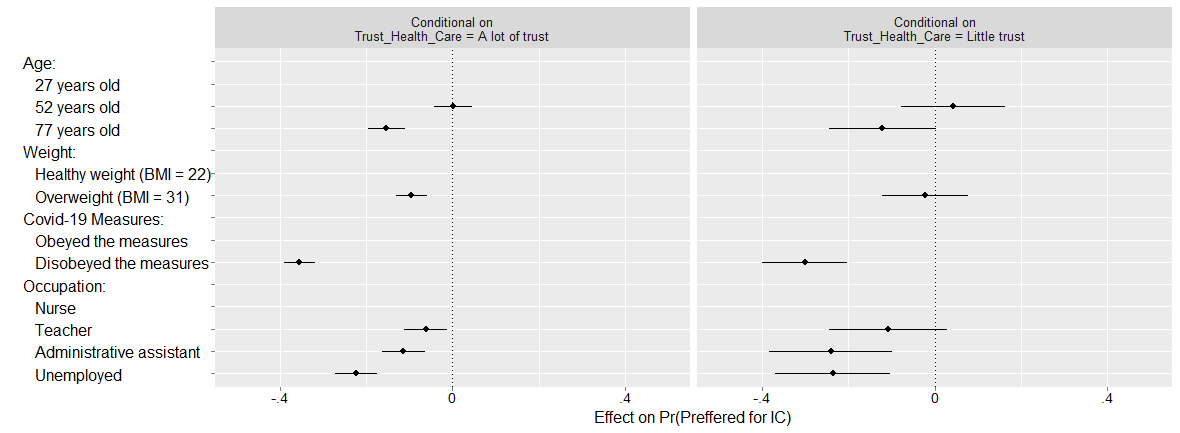


1. **Priority Access over an Early COVID-19 Vaccine**


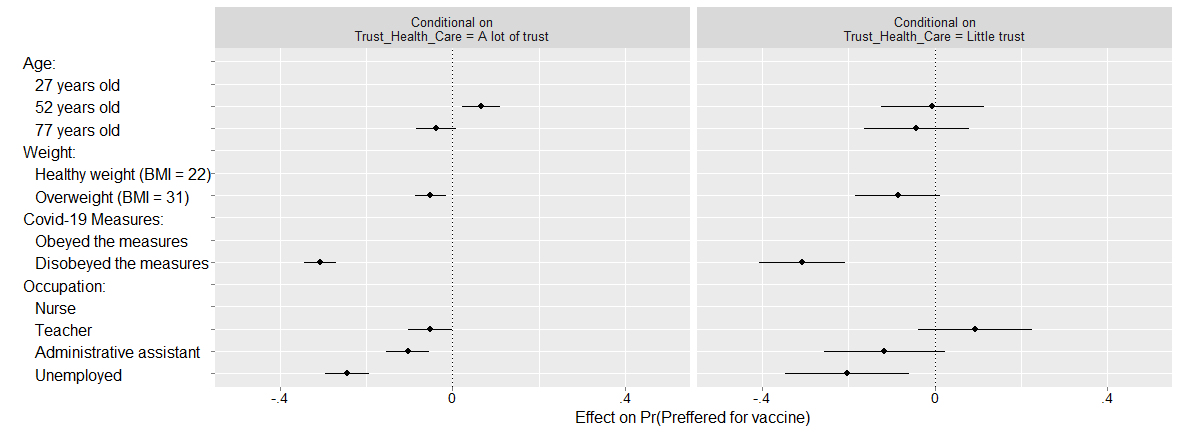

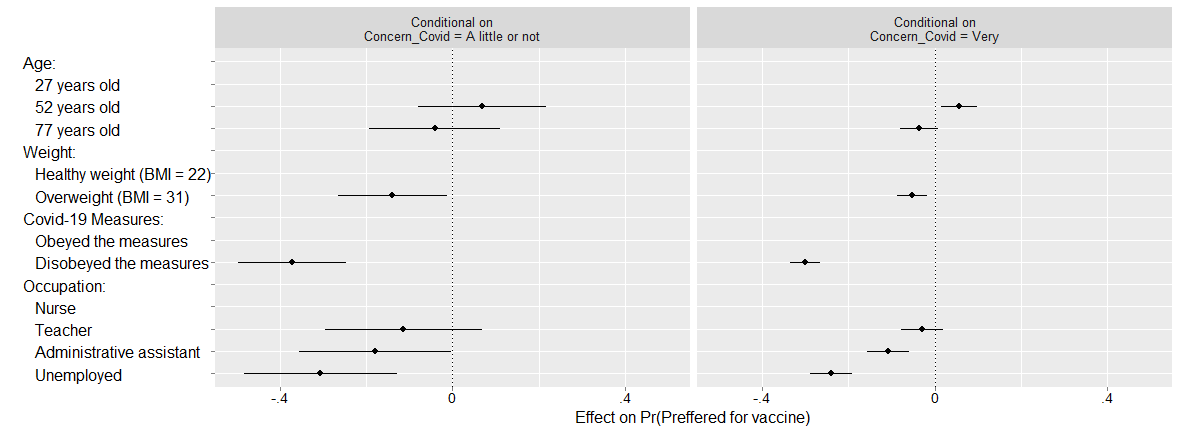

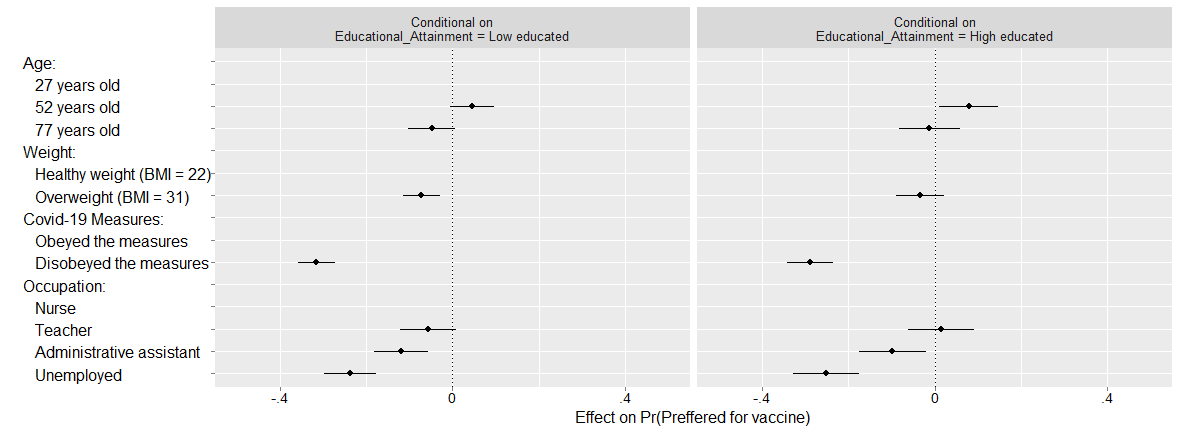

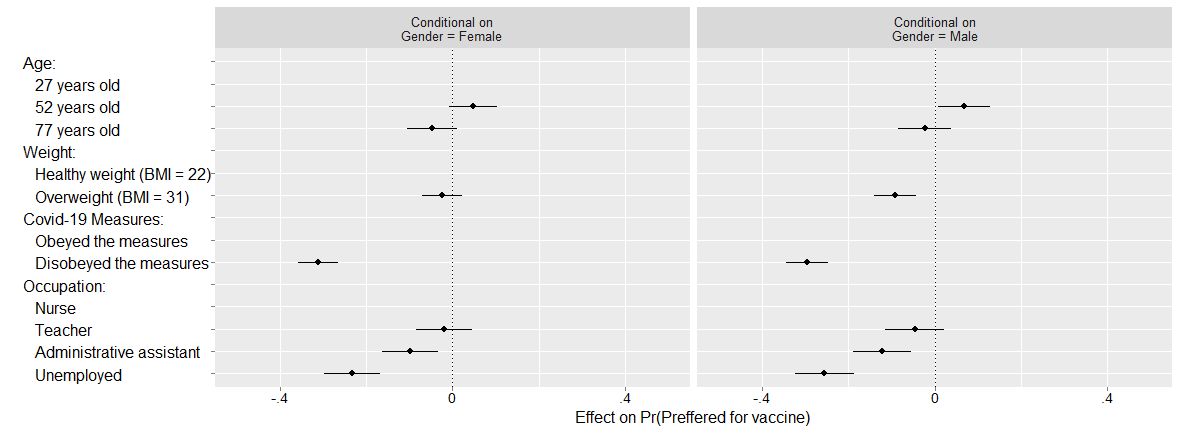

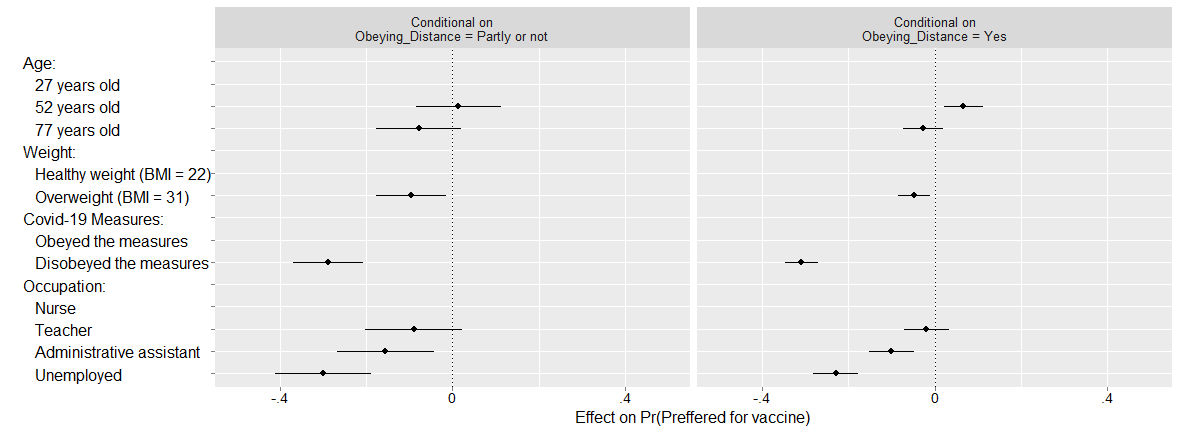

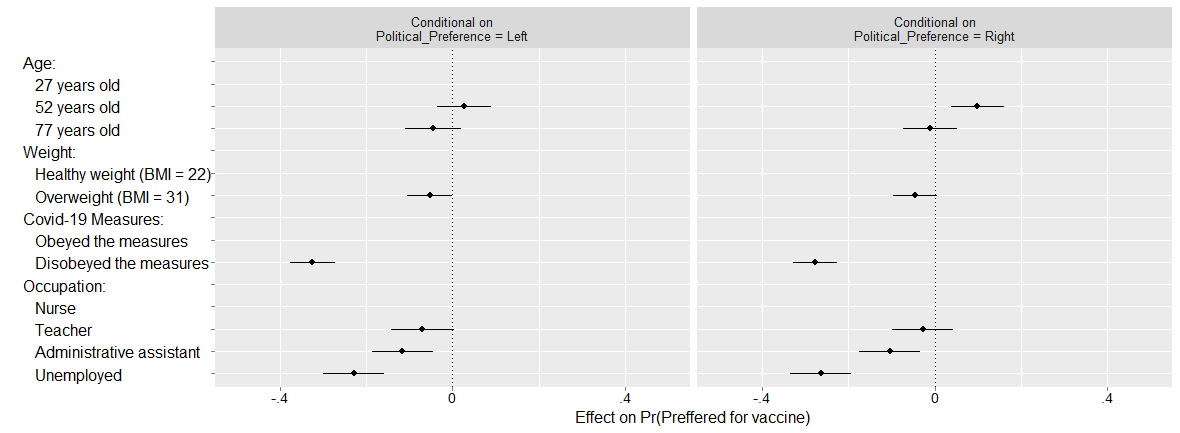

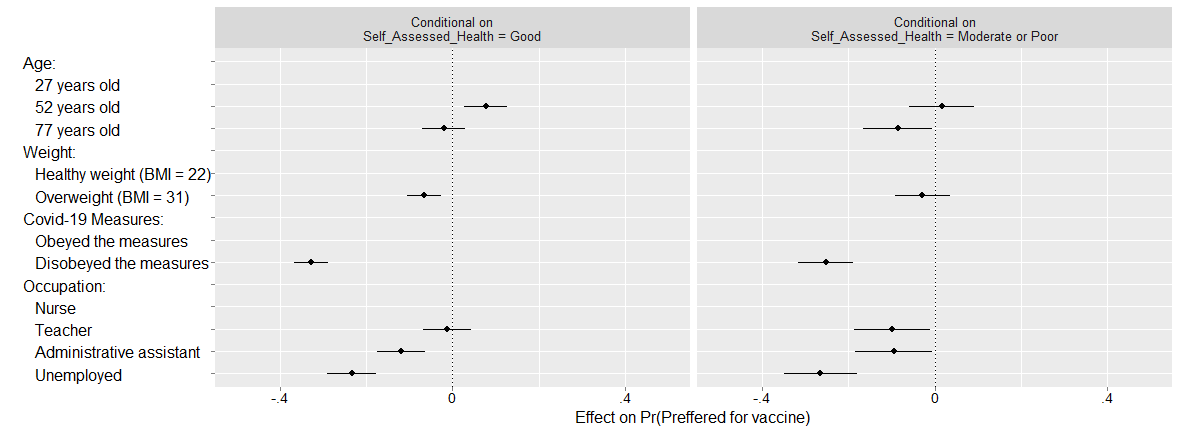

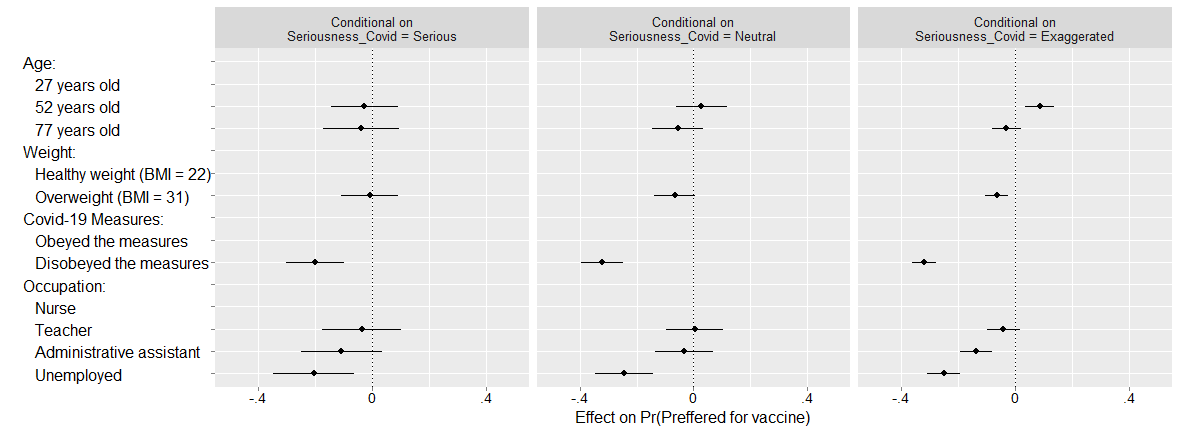


**SECTION 2: OUTPUT TABLES**

## AVERAGE MARGINAL CONDITION EFFECT INTENSIVE CARE ----------------------

------------------------------------------

Average Marginal Component Effects (AMCE):

------------------------------------------

Attribute Level Estimate Std. Err z value Pr(>|z|)

a_age_ic 2 0.00011638 0.025301 0.0045996 9.9633e-01

a_age_ic 3 -0.13566488 0.028222 -4.8071402 1.5310e-06 ***

b_weight_ic 2 -0.07392235 0.023297 -3.1730623 1.5084e-03 **

c_distance_ic 2 -0.31475766 0.019483 -16.1551301 1.0450e-58 ***

d_occupation_ic 2 -0.04796148 0.030959 -1.5491934 1.2134e-01

d_occupation_ic 3 -0.11370572 0.032259 -3.5247283 4.2392e-04 ***

d_occupation_ic 4 -0.23392881 0.029593 -7.9047714 2.6843e-15 ***

---

Number of Obs. = 2922

---

Number of Respondents = 1461

---

Signif. codes: 0 '***' 0.001 '**' 0.01 '*' 0.05

--------------------

AMCE Baseline Levels:

--------------------

Attribute Level

a_age_ic 1

b_weight_ic 1

c_distance_ic 1

d_occupation_ic 1

> #------------------------------------------------------------------------

>

> ## Interactions Age

------------------------------------------

Average Marginal Component Effects (AMCE):

------------------------------------------

Attribute Level Estimate Std. Err z value Pr(>|z|)

a_age_ic 2 0.008594 0.020881 0.41157 6.8065e-01

a_age_ic 3 -0.147540 0.020631 -7.15140 8.5896e-13 ***

b_weight_ic 2 -0.082658 0.016875 -4.89828 9.6678e-07 ***

c_distance_ic 2 -0.344929 0.016869 -20.44755 6.3169e-93 ***

d_occupation_ic 2 -0.069683 0.023610 -2.95138 3.1636e-03 **

d_occupation_ic 3 -0.138271 0.024191 -5.71572 1.0924e-08 ***

d_occupation_ic 4 -0.224375 0.023012 -9.75046 1.8363e-22 ***

---

Number of Obs. = 2922

---

Number of Respondents = 1461

---

------------------------------------------------------------

Conditional AMCE's (Generation = 16 to 40 years old):

------------------------------------------------------------

Attribute Level Estimate Std. Err z value Pr(>|z|)

a_age_ic 2 0.0102813 0.051445 0.19985 8.4160e-01

a_age_ic 3 -0.1365429 0.051641 -2.64409 8.1910e-03 **

b_weight_ic 2 -0.0465001 0.042055 -1.10570 2.6886e-01

c_distance_ic 2 -0.2853429 0.041752 -6.83416 8.2487e-12 ***

d_occupation_ic 2 0.0067313 0.061798 0.10892 9.1326e-01

d_occupation_ic 3 -0.1302753 0.059121 -2.20354 2.7557e-02 *

d_occupation_ic 4 -0.2729123 0.058362 -4.67620 2.9224e-06 ***

---

Number of Obs. = 2922

Number of Respondents = 1461

---

Signif. codes: 0 '***' 0.001 '**' 0.01 '*' 0.05

------------------------------------------------------------

Conditional AMCE's (Generation = 40 to 60 years old):

------------------------------------------------------------

Attribute Level Estimate Std. Err z value Pr(>|z|)

a_age_ic 2 -0.00014326 0.039324 -0.003643 9.9709e-01

a_age_ic 3 -0.14401773 0.039162 -3.677489 2.3554e-04 ***

b_weight_ic 2 -0.03827653 0.031741 -1.205908 2.2785e-01

c_distance_ic 2 -0.34617250 0.031717 -10.914315 9.8468e-28 ***

d_occupation_ic 2 -0.07138368 0.044462 -1.605501 1.0838e-01

d_occupation_ic 3 -0.13916412 0.046458 -2.995476 2.7402e-03 **

d_occupation_ic 4 -0.20711403 0.042645 -4.856748 1.1933e-06 ***

---

Number of Obs. = 2922

Number of Respondents = 1461

---

Signif. codes: 0 '***' 0.001 '**' 0.01 '*' 0.05

------------------------------------------------------------

Conditional AMCE's (Generation = 60 +):

------------------------------------------------------------

Attribute Level Estimate Std. Err z value Pr(>|z|)

a_age_ic 2 0.017307 0.028075 0.61647 5.3759e-01

a_age_ic 3 -0.150180 0.027542 -5.45277 4.9590e-08 ***

b_weight_ic 2 -0.120329 0.022689 -5.30340 1.1366e-07 ***

c_distance_ic 2 -0.366850 0.022580 -16.24653 2.3635e-59 ***

d_occupation_ic 2 -0.092925 0.031218 -2.97665 2.9142e-03 **

d_occupation_ic 3 -0.145480 0.032388 -4.49173 7.0646e-06 ***

d_occupation_ic 4 -0.217148 0.031064 -6.99038 2.7414e-12 ***

---

Number of Obs. = 2922

Number of Respondents = 1461

---

Signif. codes: 0 '***' 0.001 '**' 0.01 '*' 0.05

Signif. codes: 0 '***' 0.001 '**' 0.01 '*' 0.05

--------------------

AMCE Baseline Levels:

--------------------

Attribute Level

a_age_ic 1

b_weight_ic 1

c_distance_ic 1

d_occupation_ic 1

>

> #------------------------------------------------------------------------

>

> ## Interactions Distance Respondent

------------------------------------------

Average Marginal Component Effects (AMCE):

------------------------------------------

Attribute Level Estimate Std. Err z value Pr(>|z|)

a_age_ic 2 0.008594 0.020881 0.41157 6.8065e-01

a_age_ic 3 -0.147540 0.020631 -7.15140 8.5896e-13 ***

b_weight_ic 2 -0.082658 0.016875 -4.89828 9.6678e-07 ***

c_distance_ic 2 -0.344929 0.016869 -20.44755 6.3169e-93 ***

d_occupation_ic 2 -0.069683 0.023610 -2.95138 3.1636e-03 **

d_occupation_ic 3 -0.138271 0.024191 -5.71572 1.0924e-08 ***

d_occupation_ic 4 -0.224375 0.023012 -9.75046 1.8363e-22 ***

---

Number of Obs. = 2922

---

Number of Respondents = 1461

---

------------------------------------------------------------

Conditional AMCE's (ObeyingDistance = Partly or not):

------------------------------------------------------------

Attribute Level Estimate Std. Err z value Pr(>|z|)

a_age_ic 2 0.012208 0.051629 0.23646 8.1308e-01

a_age_ic 3 -0.120624 0.048803 -2.47167 1.3448e-02 *

b_weight_ic 2 -0.050178 0.041130 -1.21999 2.2247e-01

c_distance_ic 2 -0.326298 0.041359 -7.88935 3.0377e-15 ***

d_occupation_ic 2 -0.062413 0.057159 -1.09192 2.7487e-01

d_occupation_ic 3 -0.173720 0.056846 -3.05597 2.2433e-03 **

d_occupation_ic 4 -0.162950 0.058001 -2.80942 4.9631e-03 **

---

Number of Obs. = 2922

Number of Respondents = 1461

---

Signif. codes: 0 '***' 0.001 '**' 0.01 '*' 0.05

------------------------------------------------------------

Conditional AMCE's (ObeyingDistance = Yes):

------------------------------------------------------------

Attribute Level Estimate Std. Err z value Pr(>|z|)

a_age_ic 2 0.0066877 0.022873 0.29238 7.7000e-01

a_age_ic 3 -0.1543433 0.022783 -6.77443 1.2490e-11 ***

b_weight_ic 2 -0.0903219 0.018525 -4.87573 1.0841e-06 ***

c_distance_ic 2 -0.3492952 0.018496 -18.88466 1.5252e-79 ***

d_occupation_ic 2 -0.0716376 0.025962 -2.75929 5.7928e-03 **

d_occupation_ic 3 -0.1296881 0.026792 -4.84050 1.2951e-06 ***

d_occupation_ic 4 -0.2373313 0.025050 -9.47448 2.6810e-21 ***

---

Number of Obs. = 2922

Number of Respondents = 1461

---

Signif. codes: 0 '***' 0.001 '**' 0.01 '*' 0.05

Signif. codes: 0 '***' 0.001 '**' 0.01 '*' 0.05

--------------------

AMCE Baseline Levels:

--------------------

Attribute Level

a_age_ic 1

b_weight_ic 1

c_distance_ic 1

d_occupation_ic 1

> #------------------------------------------------------------------------

>

> ## Interactions Political Preference

------------------------------------------

Average Marginal Component Effects (AMCE):

------------------------------------------

Attribute Level Estimate Std. Err z value Pr(>|z|)

a_age_ic 2 0.029968 0.022214 1.3491 1.7731e-01

a_age_ic 3 -0.153574 0.021891 -7.0153 2.2947e-12 ***

b_weight_ic 2 -0.085545 0.017930 -4.7711 1.8325e-06 ***

c_distance_ic 2 -0.346988 0.017968 -19.3110 4.3395e-83 ***

d_occupation_ic 2 -0.053435 0.025084 -2.1303 3.3151e-02 *

d_occupation_ic 3 -0.122816 0.025737 -4.7719 1.8249e-06 ***

d_occupation_ic 4 -0.221909 0.024474 -9.0671 1.2226e-19 ***

---

Number of Obs. = 2560

---

Number of Respondents = 1280

---

------------------------------------------------------------

Conditional AMCE's (PoliticalPreference = Left):

------------------------------------------------------------

Attribute Level Estimate Std. Err z value Pr(>|z|)

a_age_ic 2 0.037413 0.032673 1.14507 2.5218e-01

a_age_ic 3 -0.171291 0.032144 -5.32891 9.8802e-08 ***

b_weight_ic 2 -0.089834 0.026059 -3.44732 5.6618e-04 ***

c_distance_ic 2 -0.322576 0.026080 -12.36881 3.8549e-35 ***

d_occupation_ic 2 -0.010650 0.036412 -0.29249 7.6991e-01

d_occupation_ic 3 -0.066433 0.037603 -1.76671 7.7278e-02

d_occupation_ic 4 -0.177069 0.035832 -4.94168 7.7453e-07 ***

---

Number of Obs. = 2560

Number of Respondents = 1280

---

Signif. codes: 0 '***' 0.001 '**' 0.01 '*' 0.05

------------------------------------------------------------

Conditional AMCE's (PoliticalPreference = Right):

------------------------------------------------------------

Attribute Level Estimate Std. Err z value Pr(>|z|)

a_age_ic 2 0.022013 0.030358 0.72511 4.6839e-01

a_age_ic 3 -0.140755 0.030041 -4.68536 2.7947e-06 ***

b_weight_ic 2 -0.079901 0.024762 -3.22680 1.2518e-03 **

c_distance_ic 2 -0.368621 0.024877 -14.81798 1.1211e-49 ***

d_occupation_ic 2 -0.090802 0.034680 -2.61831 8.8367e-03 **

d_occupation_ic 3 -0.174786 0.035260 -4.95709 7.1558e-07 ***

d_occupation_ic 4 -0.262555 0.033468 -7.84503 4.3286e-15 ***

---

Number of Obs. = 2560

Number of Respondents = 1280

---

Signif. codes: 0 '***' 0.001 '**' 0.01 '*' 0.05

Signif. codes: 0 '***' 0.001 '**' 0.01 '*' 0.05

--------------------

AMCE Baseline Levels:

--------------------

Attribute Level

a_age_ic 1

b_weight_ic 1

c_distance_ic 1

d_occupation_ic 1

> #------------------------------------------------------------------------

>

> ## Interactions Educational Level Respondent

------------------------------------------

Average Marginal Component Effects (AMCE):

------------------------------------------

Attribute Level Estimate Std. Err z value Pr(>|z|)

a_age_ic 2 0.010377 0.020901 0.49648 6.1956e-01

a_age_ic 3 -0.146315 0.020647 -7.08644 1.3760e-12 ***

b_weight_ic 2 -0.082543 0.016890 -4.88704 1.0236e-06 ***

c_distance_ic 2 -0.344867 0.016884 -20.42534 9.9557e-93 ***

d_occupation_ic 2 -0.069163 0.023620 -2.92818 3.4095e-03 **

d_occupation_ic 3 -0.138804 0.024205 -5.73450 9.7801e-09 ***

d_occupation_ic 4 -0.224909 0.023035 -9.76379 1.6103e-22 ***

---

Number of Obs. = 2916

---

Number of Respondents = 1458

---

------------------------------------------------------------

Conditional AMCE's (EducationalAttainment = Low educated):

------------------------------------------------------------

Attribute Level Estimate Std. Err z value Pr(>|z|)

a_age_ic 2 0.030580 0.026782 1.1418 2.5354e-01

a_age_ic 3 -0.116898 0.026603 -4.3942 1.1118e-05 ***

b_weight_ic 2 -0.090355 0.021754 -4.1535 3.2738e-05 ***

c_distance_ic 2 -0.352973 0.021693 -16.2715 1.5733e-59 ***

d_occupation_ic 2 -0.060485 0.030254 -1.9992 4.5582e-02 *

d_occupation_ic 3 -0.127536 0.031136 -4.0962 4.2004e-05 ***

d_occupation_ic 4 -0.203512 0.029518 -6.8945 5.4044e-12 ***

---

Number of Obs. = 2916

Number of Respondents = 1458

---

Signif. codes: 0 '***' 0.001 '**' 0.01 '*' 0.05

------------------------------------------------------------

Conditional AMCE's (EducationalAttainment = High educated):

------------------------------------------------------------

Attribute Level Estimate Std. Err z value Pr(>|z|)

a_age_ic 2 -0.025219 0.033412 -0.75479 4.5037e-01

a_age_ic 3 -0.193714 0.032812 -5.90368 3.5548e-09 ***

b_weight_ic 2 -0.072155 0.026859 -2.68645 7.2215e-03 **

c_distance_ic 2 -0.331252 0.026985 -12.27549 1.2265e-34 ***

d_occupation_ic 2 -0.084808 0.037883 -2.23866 2.5178e-02 *

d_occupation_ic 3 -0.159915 0.038594 -4.14349 3.4206e-05 ***

d_occupation_ic 4 -0.258855 0.036926 -7.01007 2.3820e-12 ***

---

Number of Obs. = 2916

Number of Respondents = 1458

---

Signif. codes: 0 '***' 0.001 '**' 0.01 '*' 0.05

Signif. codes: 0 '***' 0.001 '**' 0.01 '*' 0.05

--------------------

AMCE Baseline Levels:

--------------------

Attribute Level

a_age_ic 1

b_weight_ic 1

c_distance_ic 1

d_occupation_ic 1

>

> #------------------------------------------------------------------------

>

> ## Interactions Health Respondent

------------------------------------------

Average Marginal Component Effects (AMCE):

------------------------------------------

Attribute Level Estimate Std. Err z value Pr(>|z|)

a_age_ic 2 0.0082115 0.020951 0.39193 6.9511e-01

a_age_ic 3 -0.1489130 0.020748 -7.17713 7.1188e-13 ***

b_weight_ic 2 -0.0835427 0.016961 -4.92548 8.4153e-07 ***

c_distance_ic 2 -0.3442125 0.016963 -20.29195 1.5147e-91 ***

d_occupation_ic 2 -0.0705394 0.023775 -2.96695 3.0077e-03 **

d_occupation_ic 3 -0.1403611 0.024293 -5.77795 7.5616e-09 ***

d_occupation_ic 4 -0.2314432 0.023070 -10.03220 1.1004e-23 ***

---

Number of Obs. = 2886

---

Number of Respondents = 1443

---

------------------------------------------------------------

Conditional AMCE's (SelfAssessedHealth = Good):

------------------------------------------------------------

Attribute Level Estimate Std. Err z value Pr(>|z|)

a_age_ic 2 0.009561 0.025022 0.38211 7.0238e-01

a_age_ic 3 -0.148318 0.024618 -6.02477 1.6935e-09 ***

b_weight_ic 2 -0.096456 0.020187 -4.77805 1.7701e-06 ***

c_distance_ic 2 -0.346791 0.020224 -17.14717 6.5994e-66 ***

d_occupation_ic 2 -0.059937 0.028832 -2.07884 3.7632e-02 *

d_occupation_ic 3 -0.159878 0.028663 -5.57787 2.4349e-08 ***

d_occupation_ic 4 -0.222050 0.027602 -8.04463 8.6505e-16 ***

---

Number of Obs. = 2886

Number of Respondents = 1443

---

Signif. codes: 0 '***' 0.001 '**' 0.01 '*' 0.05

------------------------------------------------------------

Conditional AMCE's (SelfAssessedHealth = Moderate or Poor):

------------------------------------------------------------

Attribute Level Estimate Std. Err z value Pr(>|z|)

a_age_ic 2 0.010306 0.038436 0.26814 7.8860e-01

a_age_ic 3 -0.145804 0.038693 -3.76820 1.6443e-04 ***

b_weight_ic 2 -0.051223 0.031490 -1.62662 1.0382e-01

c_distance_ic 2 -0.337446 0.031243 -10.80064 3.4179e-27 ***

d_occupation_ic 2 -0.092205 0.042174 -2.18632 2.8792e-02 *

d_occupation_ic 3 -0.084615 0.046293 -1.82780 6.7579e-02

d_occupation_ic 4 -0.257209 0.042393 -6.06723 1.3013e-09 ***

---

Number of Obs. = 2886

Number of Respondents = 1443

---

Signif. codes: 0 '***' 0.001 '**' 0.01 '*' 0.05

Signif. codes: 0 '***' 0.001 '**' 0.01 '*' 0.05

--------------------

AMCE Baseline Levels:

--------------------

Attribute Level

a_age_ic 1

b_weight_ic 1

c_distance_ic 1

d_occupation_ic 1

> #------------------------------------------------------------------------

>

> # Interactions Trusth Health Care Respondent

------------------------------------------

Average Marginal Component Effects (AMCE):

------------------------------------------

Attribute Level Estimate Std. Err z value Pr(>|z|)

a_age_ic 2 0.0063488 0.021082 0.30114 7.6330e-01

a_age_ic 3 -0.1496879 0.020854 -7.17802 7.0726e-13 ***

b_weight_ic 2 -0.0854691 0.017056 -5.01106 5.4131e-07 ***

c_distance_ic 2 -0.3484785 0.017059 -20.42818 9.3937e-93 ***

d_occupation_ic 2 -0.0689025 0.023911 -2.88164 3.9562e-03 **

d_occupation_ic 3 -0.1302388 0.024448 -5.32719 9.9742e-08 ***

d_occupation_ic 4 -0.2254102 0.023234 -9.70160 2.9680e-22 ***

---

Number of Obs. = 2850

---

Number of Respondents = 1425

---

------------------------------------------------------------

Conditional AMCE's (TrustHealthCare = A lot of trust):

------------------------------------------------------------

Attribute Level Estimate Std. Err z value Pr(>|z|)

a_age_ic 2 0.00038287 0.022455 0.017051 9.8640e-01

a_age_ic 3 -0.15380538 0.022135 -6.948558 3.6904e-12 ***

b_weight_ic 2 -0.09558622 0.018133 -5.271368 1.3541e-07 ***

c_distance_ic 2 -0.35508918 0.018159 -19.554937 3.7447e-85 ***

d_occupation_ic 2 -0.06222790 0.025469 -2.443307 1.4553e-02 *

d_occupation_ic 3 -0.11377587 0.025972 -4.380787 1.1825e-05 ***

d_occupation_ic 4 -0.22347299 0.024705 -9.045628 1.4881e-19 ***

---

Number of Obs. = 2850

Number of Respondents = 1425

---

Signif. codes: 0 '***' 0.001 '**' 0.01 '*' 0.05

------------------------------------------------------------

Conditional AMCE's (TrustHealthCare = Little trust):

------------------------------------------------------------

Attribute Level Estimate Std. Err z value Pr(>|z|)

a_age_ic 2 0.043537 0.061366 0.70945 4.7804e-01

a_age_ic 3 -0.120861 0.063408 -1.90608 5.6640e-02

b_weight_ic 2 -0.021605 0.050783 -0.42543 6.7052e-01

c_distance_ic 2 -0.301401 0.050479 -5.97085 2.3602e-09 ***

d_occupation_ic 2 -0.107227 0.069940 -1.53313 1.2524e-01

d_occupation_ic 3 -0.240448 0.072933 -3.29681 9.7790e-04 ***

d_occupation_ic 4 -0.235924 0.067936 -3.47276 5.1514e-04 ***

---

Number of Obs. = 2850

Number of Respondents = 1425

---

Signif. codes: 0 '***' 0.001 '**' 0.01 '*' 0.05

Signif. codes: 0 '***' 0.001 '**' 0.01 '*' 0.05

--------------------

AMCE Baseline Levels:

--------------------

Attribute Level

a_age_ic 1

b_weight_ic 1

c_distance_ic 1

d_occupation_ic 1

>

> #------------------------------------------------------------------------

>

> # Interactions Gender Respondent

------------------------------------------

Average Marginal Component Effects (AMCE):

------------------------------------------

Attribute Level Estimate Std. Err z value Pr(>|z|)

a_age_ic 2 0.008594 0.020881 0.41157 6.8065e-01

a_age_ic 3 -0.147540 0.020631 -7.15140 8.5896e-13 ***

b_weight_ic 2 -0.082658 0.016875 -4.89828 9.6678e-07 ***

c_distance_ic 2 -0.344929 0.016869 -20.44755 6.3169e-93 ***

d_occupation_ic 2 -0.069683 0.023610 -2.95138 3.1636e-03 **

d_occupation_ic 3 -0.138271 0.024191 -5.71572 1.0924e-08 ***

d_occupation_ic 4 -0.224375 0.023012 -9.75046 1.8363e-22 ***

---

Number of Obs. = 2922

---

Number of Respondents = 1461

---

------------------------------------------------------------

Conditional AMCE's (Gender = Female):

------------------------------------------------------------

Attribute Level Estimate Std. Err z value Pr(>|z|)

a_age_ic 2 0.037096 0.029115 1.2741 2.0263e-01

a_age_ic 3 -0.123618 0.028737 -4.3017 1.6953e-05 ***

b_weight_ic 2 -0.039909 0.023411 -1.7047 8.8250e-02

c_distance_ic 2 -0.353118 0.023279 -15.1688 5.6903e-52 ***

d_occupation_ic 2 -0.085270 0.032794 -2.6002 9.3170e-03 **

d_occupation_ic 3 -0.149999 0.033952 -4.4179 9.9653e-06 ***

d_occupation_ic 4 -0.231010 0.031709 -7.2853 3.2102e-13 ***

---

Number of Obs. = 2922

Number of Respondents = 1461

---

Signif. codes: 0 '***' 0.001 '**' 0.01 '*' 0.05

------------------------------------------------------------

Conditional AMCE's (Gender = Male):

------------------------------------------------------------

Attribute Level Estimate Std. Err z value Pr(>|z|)

a_age_ic 2 -0.021008 0.029891 -0.70282 4.8217e-01

a_age_ic 3 -0.173680 0.029747 -5.83859 5.2643e-09 ***

b_weight_ic 2 -0.129316 0.024324 -5.31637 1.0586e-07 ***

c_distance_ic 2 -0.338236 0.024629 -13.73297 6.4437e-43 ***

d_occupation_ic 2 -0.054823 0.034165 -1.60466 1.0857e-01

d_occupation_ic 3 -0.127833 0.034446 -3.71115 2.0632e-04 ***

d_occupation_ic 4 -0.216315 0.033731 -6.41302 1.4267e-10 ***

---

Number of Obs. = 2922

Number of Respondents = 1461

---

Signif. codes: 0 '***' 0.001 '**' 0.01 '*' 0.05

Signif. codes: 0 '***' 0.001 '**' 0.01 '*' 0.05

--------------------

AMCE Baseline Levels:

--------------------

Attribute Level

a_age_ic 1

b_weight_ic 1

c_distance_ic 1

d_occupation_ic 1

> #------------------------------------------------------------------------

> # Interactions Concern COVID

------------------------------------------

Average Marginal Component Effects (AMCE):

------------------------------------------

Attribute Level Estimate Std. Err z value Pr(>|z|)

a_age_ic 2 0.008594 0.020881 0.41157 6.8065e-01

a_age_ic 3 -0.147540 0.020631 -7.15140 8.5896e-13 ***

b_weight_ic 2 -0.082658 0.016875 -4.89828 9.6678e-07 ***

c_distance_ic 2 -0.344929 0.016869 -20.44755 6.3169e-93 ***

d_occupation_ic 2 -0.069683 0.023610 -2.95138 3.1636e-03 **

d_occupation_ic 3 -0.138271 0.024191 -5.71572 1.0924e-08 ***

d_occupation_ic 4 -0.224375 0.023012 -9.75046 1.8363e-22 ***

---

Number of Obs. = 2922

---

Number of Respondents = 1461

---

------------------------------------------------------------

Conditional AMCE's (ConcernCovid = A little or not):

------------------------------------------------------------

Attribute Level Estimate Std. Err z value Pr(>|z|)

a_age_ic 2 0.038925 0.082438 0.47218 6.3680e-01

a_age_ic 3 -0.022805 0.078263 -0.29139 7.7076e-01

b_weight_ic 2 0.014245 0.066085 0.21555 8.2934e-01

c_distance_ic 2 -0.338679 0.067940 -4.98498 6.1969e-07 ***

d_occupation_ic 2 -0.090557 0.089136 -1.01594 3.0966e-01

d_occupation_ic 3 -0.169815 0.095310 -1.78171 7.4797e-02

d_occupation_ic 4 -0.180861 0.095369 -1.89644 5.7902e-02

---

Number of Obs. = 2922

Number of Respondents = 1461

---

Signif. codes: 0 '***' 0.001 '**' 0.01 '*' 0.05

------------------------------------------------------------

Conditional AMCE's (ConcernCovid = Very):

------------------------------------------------------------

Attribute Level Estimate Std. Err z value Pr(>|z|)

a_age_ic 2 0.0034111 0.021603 0.1579 8.7454e-01

a_age_ic 3 -0.1589733 0.021397 -7.4298 1.0880e-13 ***

b_weight_ic 2 -0.0908746 0.017454 -5.2066 1.9233e-07 ***

c_distance_ic 2 -0.3465256 0.017433 -19.8780 6.3041e-88 ***

d_occupation_ic 2 -0.0678977 0.024487 -2.7728 5.5573e-03 **

d_occupation_ic 3 -0.1351951 0.025003 -5.4072 6.4012e-08 ***

d_occupation_ic 4 -0.2270203 0.023689 -9.5835 9.3768e-22 ***

---

Number of Obs. = 2922

Number of Respondents = 1461

---

Signif. codes: 0 '***' 0.001 '**' 0.01 '*' 0.05

Signif. codes: 0 '***' 0.001 '**' 0.01 '*' 0.05

--------------------

AMCE Baseline Levels:

--------------------

Attribute Level

a_age_ic 1

b_weight_ic 1

c_distance_ic 1

d_occupation_ic 1

> #------------------------------------------------------------------------

>

> # Interactions opinion COVID

------------------------------------------

Average Marginal Component Effects (AMCE):

------------------------------------------

Attribute Level Estimate Std. Err z value Pr(>|z|)

a_age_ic 2 0.008594 0.020881 0.41157 6.8065e-01

a_age_ic 3 -0.147540 0.020631 -7.15140 8.5896e-13 ***

b_weight_ic 2 -0.082658 0.016875 -4.89828 9.6678e-07 ***

c_distance_ic 2 -0.344929 0.016869 -20.44755 6.3169e-93 ***

d_occupation_ic 2 -0.069683 0.023610 -2.95138 3.1636e-03 **

d_occupation_ic 3 -0.138271 0.024191 -5.71572 1.0924e-08 ***

d_occupation_ic 4 -0.224375 0.023012 -9.75046 1.8363e-22 ***

---

Number of Obs. = 2922

---

Number of Respondents = 1461

---

------------------------------------------------------------

Conditional AMCE's (SeriousnessCovid = Serious):

------------------------------------------------------------

Attribute Level Estimate Std. Err z value Pr(>|z|)

a_age_ic 2 -0.039413 0.061790 -0.63786 5.2356e-01

a_age_ic 3 -0.128788 0.061380 -2.09821 3.5886e-02 *

b_weight_ic 2 -0.091048 0.049924 -1.82373 6.8193e-02

c_distance_ic 2 -0.229782 0.050507 -4.54954 5.3764e-06 ***

d_occupation_ic 2 -0.096095 0.068519 -1.40245 1.6078e-01

d_occupation_ic 3 -0.245111 0.068588 -3.57366 3.5202e-04 ***

d_occupation_ic 4 -0.174923 0.071141 -2.45884 1.3939e-02 *

---

Number of Obs. = 2922

Number of Respondents = 1461

---

Signif. codes: 0 '***' 0.001 '**' 0.01 '*' 0.05

------------------------------------------------------------

Conditional AMCE's (SeriousnessCovid = Neutral):

------------------------------------------------------------

Attribute Level Estimate Std. Err z value Pr(>|z|)

a_age_ic 2 -0.018875 0.046847 -0.40290 6.8702e-01

a_age_ic 3 -0.144704 0.046349 -3.12208 1.7958e-03 **

b_weight_ic 2 -0.083058 0.036987 -2.24563 2.4728e-02 *

c_distance_ic 2 -0.334190 0.036838 -9.07190 1.1696e-19 ***

d_occupation_ic 2 -0.045764 0.050211 -0.91143 3.6207e-01

d_occupation_ic 3 -0.102533 0.055784 -1.83804 6.6056e-02

d_occupation_ic 4 -0.180308 0.049066 -3.67476 2.3807e-04 ***

---

Number of Obs. = 2922

Number of Respondents = 1461

---

Signif. codes: 0 '***' 0.001 '**' 0.01 '*' 0.05

------------------------------------------------------------

Conditional AMCE's (SeriousnessCovid = Exaggerated):

------------------------------------------------------------

Attribute Level Estimate Std. Err z value Pr(>|z|)

a_age_ic 2 0.026813 0.025172 1.0652 2.8679e-01

a_age_ic 3 -0.154528 0.024870 -6.2135 5.1801e-10 ***

b_weight_ic 2 -0.079315 0.020483 -3.8723 1.0782e-04 ***

c_distance_ic 2 -0.369060 0.020484 -18.0171 1.4315e-72 ***

d_occupation_ic 2 -0.073380 0.029060 -2.5251 1.1567e-02 *

d_occupation_ic 3 -0.129712 0.029225 -4.4384 9.0619e-06 ***

d_occupation_ic 4 -0.248390 0.028122 -8.8327 1.0221e-18 ***

---

Number of Obs. = 2922

Number of Respondents = 1461

---

Signif. codes: 0 '***' 0.001 '**' 0.01 '*' 0.05

Signif. codes: 0 '***' 0.001 '**' 0.01 '*' 0.05

--------------------

AMCE Baseline Levels:

--------------------

Attribute Level

a_age_ic 1

b_weight_ic 1

c_distance_ic 1

d_occupation_ic 1

| ## AVERAGE MARGINAL CONDITION EFFECT VACCIN ------------------------------------  > # AMCE Unconditional Vaccin  ------------------------------------------  Average Marginal Component Effects (AMCE):  ------------------------------------------  Attribute Level Estimate Std. Err z value Pr(>\|z\|)  a_age_vac 2 0.053869 0.027303 1.97303 4.8492e-02 *  a_age_vac 3 -0.011266 0.027853 -0.40447 6.8587e-01  b_weight_vac 2 -0.049848 0.022808 -2.18558 2.8846e-02 *  c_distance_vac 2 -0.285476 0.021352 -13.36974 9.0854e-41 ***  d_occupation_vac 2 -0.026534 0.031815 -0.83402 4.0427e-01  d_occupation_vac 3 -0.101565 0.032920 -3.08518 2.0343e-03 **  d_occupation_vac 4 -0.223090 0.031989 -6.97407 3.0789e-12 ***  ---  Number of Obs. = 2922  ---  Number of Respondents = 1461  ---  Signif. codes: 0 '***' 0.001 '**' 0.01 '*' 0.05  --------------------  AMCE Baseline Levels:  --------------------  Attribute Level  a_age_vac 1  b_weight_vac 1  c_distance_vac 1  d_occupation_vac 1  >  > #--------------------------------------------------------------------------------  >  > ## Interactions Age    ------------------------------------------  Average Marginal Component Effects (AMCE):  ------------------------------------------  Attribute Level Estimate Std. Err z value Pr(>\|z\|)  a_age_vac 2 0.057278 0.020859 2.7459 6.0343e-03 **  a_age_vac 3 -0.036277 0.021452 -1.6911 9.0824e-02  b_weight_vac 2 -0.056528 0.017203 -3.2859 1.0165e-03 **  c_distance_vac 2 -0.304133 0.017282 -17.5982 2.5421e-69 ***  d_occupation_vac 2 -0.032849 0.024163 -1.3595 1.7400e-01  d_occupation_vac 3 -0.111008 0.023990 -4.6273 3.7050e-06 ***  d_occupation_vac 4 -0.243256 0.024005 -10.1335 3.9245e-24 ***  ---  Number of Obs. = 2922  ---  Number of Respondents = 1461  ---  ------------------------------------------------------------  Conditional AMCE's (Generation = 16 to 40 years old):  ------------------------------------------------------------  Attribute Level Estimate Std. Err z value Pr(>\|z\|)  a_age_vac 2 0.133315 0.052974 2.51661 1.1849e-02 *  a_age_vac 3 0.107730 0.054112 1.99088 4.6494e-02 *  b_weight_vac 2 -0.011293 0.043014 -0.26254 7.9291e-01  c_distance_vac 2 -0.234066 0.042837 -5.46413 4.6519e-08 ***  d_occupation_vac 2 -0.034247 0.059487 -0.57571 5.6481e-01  d_occupation_vac 3 -0.076613 0.060258 -1.27143 2.0358e-01  d_occupation_vac 4 -0.261987 0.062253 -4.20845 2.5713e-05 ***  ---  Number of Obs. = 2922  Number of Respondents = 1461  ---  Signif. codes: 0 '***' 0.001 '**' 0.01 '*' 0.05  ------------------------------------------------------------  Conditional AMCE's (Generation = 40 to 60 years old):  ------------------------------------------------------------  Attribute Level Estimate Std. Err z value Pr(>\|z\|)  a_age_vac 2 0.070994 0.039377 1.80294 7.1398e-02  a_age_vac 3 -0.043191 0.039807 -1.08500 2.7792e-01  b_weight_vac 2 -0.031149 0.032329 -0.96348 3.3531e-01  c_distance_vac 2 -0.300580 0.032479 -9.25453 2.1518e-20 ***  d_occupation_vac 2 -0.013927 0.044303 -0.31435 7.5326e-01  d_occupation_vac 3 -0.065288 0.044803 -1.45721 1.4506e-01  d_occupation_vac 4 -0.214499 0.045349 -4.72994 2.2459e-06 ***  ---  Number of Obs. = 2922  Number of Respondents = 1461  ---  Signif. codes: 0 '***' 0.001 '**' 0.01 '*' 0.05  ------------------------------------------------------------  Conditional AMCE's (Generation = 60 +):  ------------------------------------------------------------  Attribute Level Estimate Std. Err z value Pr(>\|z\|)  a_age_vac 2 0.028637 0.027807 1.0298 3.0309e-01  a_age_vac 3 -0.074699 0.028998 -2.5760 9.9941e-03 **  b_weight_vac 2 -0.083110 0.023104 -3.5972 3.2167e-04 ***  c_distance_vac 2 -0.326300 0.023315 -13.9955 1.6599e-44 ***  d_occupation_vac 2 -0.045496 0.033183 -1.3711 1.7036e-01  d_occupation_vac 3 -0.151373 0.032163 -4.7065 2.5207e-06 ***  d_occupation_vac 4 -0.257321 0.031771 -8.0993 5.5254e-16 ***  ---  Number of Obs. = 2922  Number of Respondents = 1461  ---  Signif. codes: 0 '***' 0.001 '**' 0.01 '*' 0.05  Signif. codes: 0 '***' 0.001 '**' 0.01 '*' 0.05  --------------------  AMCE Baseline Levels:  --------------------  Attribute Level  a_age_vac 1  b_weight_vac 1  c_distance_vac 1  d_occupation_vac 1    > #-------------------------------------------------------------------------------  >  > ## Interactions Distance Respondent  ------------------------------------------  Average Marginal Component Effects (AMCE):  ------------------------------------------  Attribute Level Estimate Std. Err z value Pr(>\|z\|)  a_age_vac 2 0.057278 0.020859 2.7459 6.0343e-03 **  a_age_vac 3 -0.036277 0.021452 -1.6911 9.0824e-02  b_weight_vac 2 -0.056528 0.017203 -3.2859 1.0165e-03 **  c_distance_vac 2 -0.304133 0.017282 -17.5982 2.5421e-69 ***  d_occupation_vac 2 -0.032849 0.024163 -1.3595 1.7400e-01  d_occupation_vac 3 -0.111008 0.023990 -4.6273 3.7050e-06 ***  d_occupation_vac 4 -0.243256 0.024005 -10.1335 3.9245e-24 ***  ---  Number of Obs. = 2922  ---  Number of Respondents = 1461  ---  ------------------------------------------------------------  Conditional AMCE's (ObeyingDistance = Partly or not):  ------------------------------------------------------------  Attribute Level Estimate Std. Err z value Pr(>\|z\|)  a_age_vac 2 0.013485 0.050426 0.26743 7.8914e-01  a_age_vac 3 -0.078235 0.050197 -1.55857 1.1910e-01  b_weight_vac 2 -0.096287 0.040873 -2.35574 1.8486e-02 *  c_distance_vac 2 -0.288866 0.040933 -7.05714 1.6996e-12 ***  d_occupation_vac 2 -0.090246 0.057671 -1.56484 1.1762e-01  d_occupation_vac 3 -0.155538 0.057563 -2.70206 6.8910e-03 **  d_occupation_vac 4 -0.299946 0.056922 -5.26944 1.3684e-07 ***  ---  Number of Obs. = 2922  Number of Respondents = 1461  ---  Signif. codes: 0 '***' 0.001 '**' 0.01 '*' 0.05  ------------------------------------------------------------  Conditional AMCE's (ObeyingDistance = Yes):  ------------------------------------------------------------  Attribute Level Estimate Std. Err z value Pr(>\|z\|)  a_age_vac 2 0.067173 0.022942 2.92793 3.4122e-03 **  a_age_vac 3 -0.026679 0.023753 -1.12318 2.6136e-01  b_weight_vac 2 -0.047961 0.018983 -2.52648 1.1521e-02 *  c_distance_vac 2 -0.308872 0.019086 -16.18279 6.6703e-59 ***  d_occupation_vac 2 -0.018952 0.026640 -0.71139 4.7684e-01  d_occupation_vac 3 -0.100088 0.026400 -3.79119 1.4993e-04 ***  d_occupation_vac 4 -0.229483 0.026509 -8.65676 4.8538e-18 ***  ---  Number of Obs. = 2922  Number of Respondents = 1461  ---  Signif. codes: 0 '***' 0.001 '**' 0.01 '*' 0.05  Signif. codes: 0 '***' 0.001 '**' 0.01 '*' 0.05  --------------------  AMCE Baseline Levels:  --------------------  Attribute Level  a_age_vac 1  b_weight_vac 1  c_distance_vac 1  d_occupation_vac 1    > #-------------------------------------------------------------------------------  >  > ## Interactions Political Preference    ------------------------------------------  Average Marginal Component Effects (AMCE):  ------------------------------------------  Attribute Level Estimate Std. Err z value Pr(>\|z\|)  a_age_vac 2 0.064659 0.022299 2.8997 3.7353e-03 **  a_age_vac 3 -0.025996 0.022976 -1.1315 2.5786e-01  b_weight_vac 2 -0.047782 0.018414 -2.5949 9.4612e-03 **  c_distance_vac 2 -0.301158 0.018514 -16.2664 1.7100e-59 ***  d_occupation_vac 2 -0.045959 0.025977 -1.7692 7.6860e-02  d_occupation_vac 3 -0.109329 0.025492 -4.2887 1.7970e-05 ***  d_occupation_vac 4 -0.245910 0.025642 -9.5901 8.8026e-22 ***  ---  Number of Obs. = 2560  ---  Number of Respondents = 1280  ---  ------------------------------------------------------------  Conditional AMCE's (PoliticalPreference = Left):  ------------------------------------------------------------  Attribute Level Estimate Std. Err z value Pr(>\|z\|)  a_age_vac 2 0.027657 0.032052 0.86289 3.8820e-01  a_age_vac 3 -0.044480 0.033011 -1.34742 1.7784e-01  b_weight_vac 2 -0.052676 0.026424 -1.99348 4.6209e-02 *  c_distance_vac 2 -0.325100 0.026454 -12.28935 1.0333e-34 ***  d_occupation_vac 2 -0.070265 0.037442 -1.87663 6.0569e-02  d_occupation_vac 3 -0.116383 0.036376 -3.19944 1.3770e-03 **  d_occupation_vac 4 -0.229254 0.036349 -6.30710 2.8431e-10 ***  ---  Number of Obs. = 2560  Number of Respondents = 1280  ---  Signif. codes: 0 '***' 0.001 '**' 0.01 '*' 0.05  ------------------------------------------------------------  Conditional AMCE's (PoliticalPreference = Right):  ------------------------------------------------------------  Attribute Level Estimate Std. Err z value Pr(>\|z\|)  a_age_vac 2 0.099408 0.031123 3.19403 1.4030e-03 **  a_age_vac 3 -0.010649 0.032027 -0.33251 7.3951e-01  b_weight_vac 2 -0.045036 0.025711 -1.75159 7.9844e-02  c_distance_vac 2 -0.277769 0.026007 -10.68064 1.2540e-26 ***  d_occupation_vac 2 -0.027248 0.036048 -0.75588 4.4972e-01  d_occupation_vac 3 -0.104179 0.035850 -2.90597 3.6611e-03 **  d_occupation_vac 4 -0.264077 0.036140 -7.30707 2.7303e-13 ***  ---  Number of Obs. = 2560  Number of Respondents = 1280  ---  Signif. codes: 0 '***' 0.001 '**' 0.01 '*' 0.05  Signif. codes: 0 '***' 0.001 '**' 0.01 '*' 0.05  --------------------  AMCE Baseline Levels:  --------------------  Attribute Level  a_age_vac 1  b_weight_vac 1  c_distance_vac 1  d_occupation_vac 1    > #-------------------------------------------------------------------------------  >  > ## Interactions Educational Level Respondent  ------------------------------------------  Average Marginal Component Effects (AMCE):  ------------------------------------------  Attribute Level Estimate Std. Err z value Pr(>\|z\|)  a_age_vac 2 0.057156 0.020945 2.7288 6.3567e-03 **  a_age_vac 3 -0.034884 0.022128 -1.5764 1.1493e-01  b_weight_vac 2 -0.057455 0.017267 -3.3274 8.7663e-04 ***  c_distance_vac 2 -0.305601 0.016715 -18.2834 1.1230e-74 ***  d_occupation_vac 2 -0.029193 0.025230 -1.1571 2.4724e-01  d_occupation_vac 3 -0.110748 0.024669 -4.4894 7.1423e-06 ***  d_occupation_vac 4 -0.242042 0.024305 -9.9584 2.3167e-23 ***  ---  Number of Obs. = 2916  ---  Number of Respondents = 1458  ---  ------------------------------------------------------------  Conditional AMCE's (EducationalAttainment = Low educated):  ------------------------------------------------------------  Attribute Level Estimate Std. Err z value Pr(>\|z\|)  a_age_vac 2 0.045269 0.026181 1.7290 8.3801e-02  a_age_vac 3 -0.048651 0.028300 -1.7191 8.5591e-02  b_weight_vac 2 -0.072789 0.021903 -3.3232 8.8997e-04 ***  c_distance_vac 2 -0.315573 0.021333 -14.7928 1.6308e-49 ***  d_occupation_vac 2 -0.057137 0.033132 -1.7245 8.4613e-02  d_occupation_vac 3 -0.119374 0.031523 -3.7869 1.5256e-04 ***  d_occupation_vac 4 -0.238166 0.031127 -7.6514 1.9877e-14 ***  ---  Number of Obs. = 2916  Number of Respondents = 1458  ---  Signif. codes: 0 '***' 0.001 '**' 0.01 '*' 0.05  ------------------------------------------------------------  Conditional AMCE's (EducationalAttainment = High educated):  ------------------------------------------------------------  Attribute Level Estimate Std. Err z value Pr(>\|z\|)  a_age_vac 2 0.079545 0.034710 2.29174 2.1921e-02 *  a_age_vac 3 -0.011921 0.035686 -0.33405 7.3834e-01  b_weight_vac 2 -0.033283 0.028127 -1.18330 2.3669e-01  c_distance_vac 2 -0.289176 0.026908 -10.74702 6.1209e-27 ***  d_occupation_vac 2 0.015467 0.038799 0.39863 6.9016e-01  d_occupation_vac 3 -0.098304 0.039574 -2.48406 1.2989e-02 *  d_occupation_vac 4 -0.251628 0.039241 -6.41235 1.4329e-10 ***  ---  Number of Obs. = 2916  Number of Respondents = 1458  ---  Signif. codes: 0 '***' 0.001 '**' 0.01 '*' 0.05  Signif. codes: 0 '***' 0.001 '**' 0.01 '*' 0.05  --------------------  AMCE Baseline Levels:  --------------------  Attribute Level  a_age_vac 1  b_weight_vac 1  c_distance_vac 1  d_occupation_vac 1  > #-------------------------------------------------------------------------------  >  > ## Interactions Health Respondent  ------------------------------------------  Average Marginal Component Effects (AMCE):  ------------------------------------------  Attribute Level Estimate Std. Err z value Pr(>\|z\|)  a_age_vac 2 0.061000 0.020953 2.9114 3.5987e-03 **  a_age_vac 3 -0.037245 0.021579 -1.7259 8.4357e-02  b_weight_vac 2 -0.056445 0.017291 -3.2644 1.0968e-03 **  c_distance_vac 2 -0.306605 0.017371 -17.6502 1.0145e-69 ***  d_occupation_vac 2 -0.036223 0.024221 -1.4955 1.3477e-01  d_occupation_vac 3 -0.113012 0.024081 -4.6929 2.6929e-06 ***  d_occupation_vac 4 -0.242360 0.024138 -10.0407 1.0094e-23 ***  ---  Number of Obs. = 2886  ---  Number of Respondents = 1443  ---  ------------------------------------------------------------  Conditional AMCE's (SelfAssessedHealth = Good):  ------------------------------------------------------------  Attribute Level Estimate Std. Err z value Pr(>\|z\|)  a_age_vac 2 0.076664 0.025027 3.06331 2.1890e-03 **  a_age_vac 3 -0.020527 0.025404 -0.80801 4.1908e-01  b_weight_vac 2 -0.066272 0.020517 -3.23018 1.2371e-03 **  c_distance_vac 2 -0.328519 0.020521 -16.00877 1.1099e-57 ***  d_occupation_vac 2 -0.012895 0.028746 -0.44860 6.5372e-01  d_occupation_vac 3 -0.119268 0.028532 -4.18017 2.9130e-05 ***  d_occupation_vac 4 -0.233838 0.028945 -8.07875 6.5431e-16 ***  ---  Number of Obs. = 2886  Number of Respondents = 1443  ---  Signif. codes: 0 '***' 0.001 '**' 0.01 '*' 0.05  ------------------------------------------------------------  Conditional AMCE's (SelfAssessedHealth = Moderate or Poor):  ------------------------------------------------------------  Attribute Level Estimate Std. Err z value Pr(>\|z\|)  a_age_vac 2 0.016334 0.038357 0.42584 6.7022e-01  a_age_vac 3 -0.085220 0.040996 -2.07875 3.7641e-02 *  b_weight_vac 2 -0.028935 0.032525 -0.88964 3.7366e-01  c_distance_vac 2 -0.252852 0.032592 -7.75807 8.6234e-15 ***  d_occupation_vac 2 -0.098029 0.044911 -2.18273 2.9056e-02 *  d_occupation_vac 3 -0.094869 0.045217 -2.09806 3.5900e-02 *  d_occupation_vac 4 -0.264883 0.043564 -6.08032 1.1994e-09 ***  ---  Number of Obs. = 2886  Number of Respondents = 1443  ---  Signif. codes: 0 '***' 0.001 '**' 0.01 '*' 0.05  Signif. codes: 0 '***' 0.001 '**' 0.01 '*' 0.05  --------------------  AMCE Baseline Levels:  --------------------  Attribute Level  a_age_vac 1  b_weight_vac 1  c_distance_vac 1  d_occupation_vac 1  > #-------------------------------------------------------------------------------  >  > # Interactions Trusth Health Care Respondent  >  ------------------------------------------  Average Marginal Component Effects (AMCE):  ------------------------------------------  Attribute Level Estimate Std. Err z value Pr(>\|z\|)  a_age_vac 2 0.058009 0.021089 2.7508 5.9457e-03 **  a_age_vac 3 -0.038338 0.021691 -1.7675 7.7142e-02  b_weight_vac 2 -0.055679 0.017400 -3.2000 1.3745e-03 **  c_distance_vac 2 -0.306812 0.017490 -17.5422 6.8266e-69 ***  d_occupation_vac 2 -0.033132 0.024399 -1.3579 1.7449e-01  d_occupation_vac 3 -0.107279 0.024209 -4.4313 9.3670e-06 ***  d_occupation_vac 4 -0.240466 0.024352 -9.8748 5.3543e-23 ***  ---  Number of Obs. = 2850  ---  Number of Respondents = 1425  ---  ------------------------------------------------------------  Conditional AMCE's (TrustHealthCare = A lot of trust):  ------------------------------------------------------------  Attribute Level Estimate Std. Err z value Pr(>\|z\|)  a_age_vac 2 0.066009 0.022488 2.9354 3.3316e-03 **  a_age_vac 3 -0.038167 0.023215 -1.6441 1.0017e-01  b_weight_vac 2 -0.051324 0.018592 -2.7606 5.7691e-03 **  c_distance_vac 2 -0.307127 0.018647 -16.4709 5.9356e-61 ***  d_occupation_vac 2 -0.051531 0.026208 -1.9662 4.9272e-02 *  d_occupation_vac 3 -0.104135 0.025741 -4.0454 5.2232e-05 ***  d_occupation_vac 4 -0.244157 0.025828 -9.4531 3.2902e-21 ***  ---  Number of Obs. = 2850  Number of Respondents = 1425  ---  Signif. codes: 0 '***' 0.001 '**' 0.01 '*' 0.05  ------------------------------------------------------------  Conditional AMCE's (TrustHealthCare = Little trust):  ------------------------------------------------------------  Attribute Level Estimate Std. Err z value Pr(>\|z\|)  a_age_vac 2 -0.0050186 0.061374 -0.081771 9.3483e-01  a_age_vac 3 -0.0417851 0.062191 -0.671879 5.0166e-01  b_weight_vac 2 -0.0852132 0.050060 -1.702215 8.8715e-02  c_distance_vac 2 -0.3075689 0.051060 -6.023698 1.7048e-09 ***  d_occupation_vac 2 0.0936060 0.067845 1.379697 1.6768e-01  d_occupation_vac 3 -0.1166030 0.071618 -1.628124 1.0350e-01  d_occupation_vac 4 -0.2032607 0.073738 -2.756511 5.8422e-03 **  ---  Number of Obs. = 2850  Number of Respondents = 1425  ---  Signif. codes: 0 '***' 0.001 '**' 0.01 '*' 0.05  Signif. codes: 0 '***' 0.001 '**' 0.01 '*' 0.05  --------------------  AMCE Baseline Levels:  --------------------  Attribute Level  a_age_vac 1  b_weight_vac 1  c_distance_vac 1  d_occupation_vac 1  >  > #-------------------------------------------------------------------------------  > # Interactions Gender Respondent  ------------------------------------------  Average Marginal Component Effects (AMCE):  ------------------------------------------  Attribute Level Estimate Std. Err z value Pr(>\|z\|)  a_age_vac 2 0.057278 0.020859 2.7459 6.0343e-03 **  a_age_vac 3 -0.036277 0.021452 -1.6911 9.0824e-02  b_weight_vac 2 -0.056528 0.017203 -3.2859 1.0165e-03 **  c_distance_vac 2 -0.304133 0.017282 -17.5982 2.5421e-69 ***  d_occupation_vac 2 -0.032849 0.024163 -1.3595 1.7400e-01  d_occupation_vac 3 -0.111008 0.023990 -4.6273 3.7050e-06 ***  d_occupation_vac 4 -0.243256 0.024005 -10.1335 3.9245e-24 ***  ---  Number of Obs. = 2922  ---  Number of Respondents = 1461  ---  ------------------------------------------------------------  Conditional AMCE's (Gender = Female):  ------------------------------------------------------------  Attribute Level Estimate Std. Err z value Pr(>\|z\|)  a_age_vac 2 0.047548 0.028956 1.64207 1.0058e-01  a_age_vac 3 -0.047884 0.029290 -1.63484 1.0208e-01  b_weight_vac 2 -0.024621 0.023786 -1.03509 3.0063e-01  c_distance_vac 2 -0.312414 0.023902 -13.07036 4.8637e-39 ***  d_occupation_vac 2 -0.020202 0.033175 -0.60894 5.4256e-01  d_occupation_vac 3 -0.098487 0.033472 -2.94239 3.2569e-03 **  d_occupation_vac 4 -0.233434 0.033198 -7.03148 2.0436e-12 ***  ---  Number of Obs. = 2922  Number of Respondents = 1461  ---  Signif. codes: 0 '***' 0.001 '**' 0.01 '*' 0.05  ------------------------------------------------------------  Conditional AMCE's (Gender = Male):  ------------------------------------------------------------  Attribute Level Estimate Std. Err z value Pr(>\|z\|)  a_age_vac 2 0.068401 0.030215 2.26382 2.3585e-02 *  a_age_vac 3 -0.022603 0.031595 -0.71541 4.7436e-01  b_weight_vac 2 -0.091627 0.024958 -3.67123 2.4139e-04 ***  c_distance_vac 2 -0.296287 0.025076 -11.81543 3.2488e-32 ***  d_occupation_vac 2 -0.046148 0.035374 -1.30458 1.9203e-01  d_occupation_vac 3 -0.122689 0.034434 -3.56306 3.6656e-04 ***  d_occupation_vac 4 -0.255860 0.034900 -7.33118 2.2814e-13 ***  ---  Number of Obs. = 2922  Number of Respondents = 1461  ---  Signif. codes: 0 '***' 0.001 '**' 0.01 '*' 0.05  Signif. codes: 0 '***' 0.001 '**' 0.01 '*' 0.05  --------------------  AMCE Baseline Levels:  --------------------  Attribute Level  a_age_vac 1  b_weight_vac 1  c_distance_vac 1  d_occupation_vac 1  >  > #-------------------------------------------------------------------------------  >  > # Interactions Concern COVID  ------------------------------------------  Average Marginal Component Effects (AMCE):  ------------------------------------------  Attribute Level Estimate Std. Err z value Pr(>\|z\|)  a_age_vac 2 0.057278 0.020859 2.7459 6.0343e-03 **  a_age_vac 3 -0.036277 0.021452 -1.6911 9.0824e-02  b_weight_vac 2 -0.056528 0.017203 -3.2859 1.0165e-03 **  c_distance_vac 2 -0.304133 0.017282 -17.5982 2.5421e-69 ***  d_occupation_vac 2 -0.032849 0.024163 -1.3595 1.7400e-01  d_occupation_vac 3 -0.111008 0.023990 -4.6273 3.7050e-06 ***  d_occupation_vac 4 -0.243256 0.024005 -10.1335 3.9245e-24 ***  ---  Number of Obs. = 2922  ---  Number of Respondents = 1461  ---  ------------------------------------------------------------  Conditional AMCE's (ConcernCovid = A little or not):  ------------------------------------------------------------  Attribute Level Estimate Std. Err z value Pr(>\|z\|)  a_age_vac 2 0.068929 0.075802 0.90933 3.6318e-01  a_age_vac 3 -0.041438 0.077350 -0.53572 5.9215e-01  b_weight_vac 2 -0.139371 0.064060 -2.17563 2.9583e-02 *  c_distance_vac 2 -0.372612 0.064207 -5.80333 6.5013e-09 ***  d_occupation_vac 2 -0.113785 0.093062 -1.22269 2.2145e-01  d_occupation_vac 3 -0.179463 0.090260 -1.98829 4.6780e-02 *  d_occupation_vac 4 -0.306744 0.090553 -3.38746 7.0544e-04 ***  ---  Number of Obs. = 2922  Number of Respondents = 1461  ---  Signif. codes: 0 '***' 0.001 '**' 0.01 '*' 0.05  ------------------------------------------------------------  Conditional AMCE's (ConcernCovid = Very):  ------------------------------------------------------------  Attribute Level Estimate Std. Err z value Pr(>\|z\|)  a_age_vac 2 0.056499 0.021715 2.6019 9.2714e-03 **  a_age_vac 3 -0.035443 0.022356 -1.5854 1.1287e-01  b_weight_vac 2 -0.051381 0.017913 -2.8683 4.1269e-03 **  c_distance_vac 2 -0.299873 0.017971 -16.6865 1.6444e-62 ***  d_occupation_vac 2 -0.028292 0.025066 -1.1287 2.5903e-01  d_occupation_vac 3 -0.107050 0.024982 -4.2850 1.8270e-05 ***  d_occupation_vac 4 -0.239438 0.024940 -9.6007 7.9425e-22 ***  ---  Number of Obs. = 2922  Number of Respondents = 1461  ---  Signif. codes: 0 '***' 0.001 '**' 0.01 '*' 0.05  Signif. codes: 0 '***' 0.001 '**' 0.01 '*' 0.05  --------------------  AMCE Baseline Levels:  --------------------  Attribute Level  a_age_vac 1  b_weight_vac 1  c_distance_vac 1  d_occupation_vac 1    > #-------------------------------------------------------------------------------  > # Interactions opinion COVID  ------------------------------------------  Average Marginal Component Effects (AMCE):  ------------------------------------------  Attribute Level Estimate Std. Err z value Pr(>\|z\|)  a_age_vac 2 0.057278 0.020859 2.7459 6.0343e-03 **  a_age_vac 3 -0.036277 0.021452 -1.6911 9.0824e-02  b_weight_vac 2 -0.056528 0.017203 -3.2859 1.0165e-03 **  c_distance_vac 2 -0.304133 0.017282 -17.5982 2.5421e-69 ***  d_occupation_vac 2 -0.032849 0.024163 -1.3595 1.7400e-01  d_occupation_vac 3 -0.111008 0.023990 -4.6273 3.7050e-06 ***  d_occupation_vac 4 -0.243256 0.024005 -10.1335 3.9245e-24 ***  ---  Number of Obs. = 2922  ---  Number of Respondents = 1461  ---  ------------------------------------------------------------  Conditional AMCE's (SeriousnessCovid = Serious):  ------------------------------------------------------------  Attribute Level Estimate Std. Err z value Pr(>\|z\|)  a_age_vac 2 -0.0271568 0.060371 -0.44983 0.65283385  a_age_vac 3 -0.0387327 0.068537 -0.56514 0.57197909  b_weight_vac 2 -0.0088104 0.051560 -0.17088 0.86431946  c_distance_vac 2 -0.2020790 0.052068 -3.88110 0.00010399 ***  d_occupation_vac 2 -0.0368449 0.070904 -0.51964 0.60331208  d_occupation_vac 3 -0.1076133 0.071667 -1.50157 0.13320722  d_occupation_vac 4 -0.2054525 0.072957 -2.81609 0.00486119 **  ---  Number of Obs. = 2922  Number of Respondents = 1461  ---  Signif. codes: 0 '***' 0.001 '**' 0.01 '*' 0.05  ------------------------------------------------------------  Conditional AMCE's (SeriousnessCovid = Neutral):  ------------------------------------------------------------  Attribute Level Estimate Std. Err z value Pr(>\|z\|)  a_age_vac 2 0.0267734 0.045684 0.586055 5.5784e-01  a_age_vac 3 -0.0547566 0.045601 -1.200788 2.2983e-01  b_weight_vac 2 -0.0654588 0.036891 -1.774395 7.5998e-02  c_distance_vac 2 -0.3216447 0.037207 -8.644719 5.3938e-18 ***  d_occupation_vac 2 0.0041407 0.051475 0.080441 9.3589e-01  d_occupation_vac 3 -0.0339998 0.052106 -0.652514 5.1407e-01  d_occupation_vac 4 -0.2442794 0.051396 -4.752893 2.0053e-06 ***  ---  Number of Obs. = 2922  Number of Respondents = 1461  ---  Signif. codes: 0 '***' 0.001 '**' 0.01 '*' 0.05  ------------------------------------------------------------  Conditional AMCE's (SeriousnessCovid = Exaggerated):  ------------------------------------------------------------  Attribute Level Estimate Std. Err z value Pr(>\|z\|)  a_age_vac 2 0.087432 0.025527 3.4251 6.1450e-04 ***  a_age_vac 3 -0.029465 0.026047 -1.1312 2.5797e-01  b_weight_vac 2 -0.063581 0.021036 -3.0225 2.5068e-03 **  c_distance_vac 2 -0.319583 0.021122 -15.1303 1.0227e-51 ***  d_occupation_vac 2 -0.041149 0.029642 -1.3882 1.6508e-01  d_occupation_vac 3 -0.136006 0.029248 -4.6500 3.3188e-06 ***  d_occupation_vac 4 -0.249383 0.029231 -8.5316 1.4439e-17 ***  ---  Number of Obs. = 2922  Number of Respondents = 1461  ---  Signif. codes: 0 '***' 0.001 '**' 0.01 '*' 0.05  Signif. codes: 0 '***' 0.001 '**' 0.01 '*' 0.05  --------------------  AMCE Baseline Levels:  --------------------  Attribute Level  a_age_vac 1  b_weight_vac 1  c_distance_vac 1  d_occupation_vac 1 |
| --- |
|  |
|  |

**SECTION 3: SYNTAX**

## Load packages ---------------------------------------------------------------

library(dplyr)

library(tidyr)

library(survey)

library(cjoint)

library(ggplot2)

library(haven)

library(data.table)

library(purrr)

library(gmodels)

library(naniar)

## Load data -------------------------------------------------------------------

Between_Corona_October2020_weighted <- read_sav("corona.sav")

## rename column names ---------------------------------------------------------

EVS <- rename(Between_Corona_October2020_weighted, c('ageA' = 'arandom1',

'ageB'= 'arandom2',

'weightA' = 'brandom1',

'weightB' = 'brandom2',

'distanceA' = 'crandom1',

'distanceB' = 'crandom2',

'occupationA' = 'drandom1',

'occupationB' = 'drandom2',

'ageC' = 'grandom1',

'ageD'= 'grandom2',

'weightC' = 'hrandom1',

'weightD' = 'hrandom2',

'distanceC' = 'irandom1',

'distanceD' = 'irandom2',

'occupationC' = 'jrandom1',

'occupationD' = 'jrandom2',

'Generation' = 'leeftijd',

'Political_Preference' = 'Q31',

'Obeying_Distance' = 'corona7',

'Educational_Attainment' = 'oplcat',

'Self_Assessed_Health' = 'Q3',

'Trust_Health_Care'= 'Q38L',

'Gender' = 'Sex',

'Concern_Covid' = 'corona1',

'Seriousness_Covid' = 'corona9'))

## replace the colums into rows ------------------------------------------------

## duplicate rows

EVS_dupl <- rbindlist(list(EVS, EVS))[order(ID)]

## create condition column

EVS_cond <- EVS

condA <- 'A'

condB <- 'B'

EVS_cond <- cbind(EVS_cond, condA)

EVS_cond <- cbind(EVS_cond, condB)

## combine columns under each other

EVS_dupl$a_age_ic <- c(do.call(rbind, lapply(EVS[5:6], as.character)))

EVS_dupl$b_weight_ic <- c(do.call(rbind, lapply(EVS[7:8], as.character)))

EVS_dupl$c_distance_ic <- c(do.call(rbind, lapply(EVS[9:10], as.character)))

EVS_dupl$d_occupation_ic <- c(do.call(rbind, lapply(EVS [11:12], as.character)))

EVS_dupl$a_age_vac <- c(do.call(rbind, lapply(EVS[13:14], as.character)))

EVS_dupl$b_weight_vac <- c(do.call(rbind, lapply(EVS[15:16], as.character)))

EVS_dupl$c_distance_vac <- c(do.call(rbind, lapply(EVS[17:18], as.character)))

EVS_dupl$d_occupation_vac <- c(do.call(rbind, lapply(EVS[19:20], as.character)))

EVS_dupl$condition <- c(do.call(rbind, lapply(EVS_cond[130:131], as.character)))

EVS_dupl$Political_Preference <- as.numeric(EVS_dupl$Political_Preference)

EVS_dupl$Obeying_Distance <- as.factor(EVS_dupl$Obeying_Distance)

EVS_dupl$Educational_Attainment <- as.numeric(EVS_dupl$Educational_Attainment)

EVS_dupl$Self_Assessed_Health <- as.numeric(EVS_dupl$Self_Assessed_Health)

EVS_dupl$Trust_Health_Care <- as.numeric(EVS_dupl$Trust_Health_Care)

EVS_dupl$Gender <- as.numeric(EVS_dupl$Gender)

EVS_dupl$Concern_Covid <- as.numeric(EVS_dupl$Concern_Covid)

EVS_dupl$Seriousness_Covid <-as.numeric(EVS_dupl$Seriousness_Covid)

## assign choice of participant to condition

## vignette_versie = the chosen vignette, condition = whether the row is A or B

EVS_dupl$outcome_IC[EVS_dupl$vignet_versie1== 1 & EVS_dupl$condition=="B"] <- 0

EVS_dupl$outcome_IC[EVS_dupl$vignet_versie1== 1 & EVS_dupl$condition=="A"] <- 1

EVS_dupl$outcome_IC[EVS_dupl$vignet_versie1== 2 & EVS_dupl$condition=="A"] <- 0

EVS_dupl$outcome_IC[EVS_dupl$vignet_versie1== 2 & EVS_dupl$condition=="B"] <- 1

EVS_dupl$outcome_vac[EVS_dupl$vignet_versie2== 1 & EVS_dupl$condition=='B'] <- 0

EVS_dupl$outcome_vac[EVS_dupl$vignet_versie2== 1 & EVS_dupl$condition=='A'] <- 1

EVS_dupl$outcome_vac[EVS_dupl$vignet_versie2== 2 & EVS_dupl$condition=='A'] <- 0

EVS_dupl$outcome_vac[EVS_dupl$vignet_versie2== 2 & EVS_dupl$condition=='B'] <- 1

## create dataframe with only the needed data ----------------------------------

df <- data.frame(EVS_dupl$ID, EVS_dupl$Generation, EVS_dupl$Political_Preference,

EVS_dupl$Obeying_Distance, EVS_dupl$Educational_Attainment,

EVS_dupl$a_age_ic, EVS_dupl$b_weight_ic, EVS_dupl$c_distance_ic,

EVS_dupl$d_occupation_ic, EVS_dupl$a_age_vac, EVS_dupl$b_weight_vac,

EVS_dupl$c_distance_vac, EVS_dupl$d_occupation_vac,

EVS_dupl$outcome_IC, EVS_dupl$outcome_vac, EVS_dupl$weight_t,

EVS_dupl$Self_Assessed_Health, EVS_dupl$Trust_Health_Care,

EVS_dupl$Gender, EVS_dupl$Concern_Covid, EVS_dupl$Seriousness_Covid)

names(df) <- c('ID', 'Generation', 'Political_Preference', 'Obeying_Distance',

'Educational_Attainment', 'a_age_ic', 'b_weight_ic', 'c_distance_ic',

'd_occupation_ic', 'a_age_vac', 'b_weight_vac', 'c_distance_vac',

'd_occupation_vac', 'outcome_ic', 'outcome_vac', 'data_weights',

'Self_Assessed_Health', 'Trust_Health_Care', 'Gender',

'Concern_Covid', 'Seriousness_Covid')

# removing the missing values

df <- df %>% drop_na(outcome_ic) ## removing these rows

df <- df %>% drop_na(outcome_vac)

df <- replace_with_na(df, replace = list(Political_Preference = -9)) ## replacing the value with NA

df <- replace_with_na(df, replace = list(Political_Preference = -8))

df <- replace_with_na(df, replace = list(Educational_Attainment = 9))

df <- replace_with_na(df, replace = list(Self_Assessed_Health=-9))

df <- replace_with_na(df, replace = list(Self_Assessed_Health=-8))

df <- replace_with_na(df, replace = list(Trust_Health_Care=-9))

df <- replace_with_na(df, replace = list(Trust_Health_Care=-8))

## Descriptives ---------------------------------------------------------------

summary(df$Generation)

summary(df$Political_Preference)

summary(df$Educational_Attainment)

summary(df$Obeying_Distance)

summary(df$Self_Assessed_Health)

summary(df$Trust_Health_Care)

summary(df$Gender)

summary(df$Concern_Covid)

summary(df$Seriousness_Covid)

## split into intervals + rename values ----------------------------------------

df$Generation <- cut(df$Generation, breaks=c(16,40, 60, 96), right = FALSE,

labels= c('16 to 40 years old', '40 to 60 years old', '60 +')) ## 40=40-60

df$Political_Preference <- cut(df$Political_Preference, breaks=c(1, 6, 11),

labels = c('Left', 'Right'), right=FALSE)

df$Educational_Attainment <- cut(df$Educational_Attainment, breaks= c(1,5,7),

right=FALSE, labels = c('Low educated', 'High educated'))

df$Obeying_Distance2[df$Obeying_Distance == 1] <- 'Yes'

df$Obeying_Distance2[df$Obeying_Distance == 2] <- 'Partly or not'

df$Obeying_Distance2[df$Obeying_Distance == 3] <- 'Partly or not'

df$Obeying_Distance<- as.factor(df$Obeying_Distance2)

df$Self_Assessed_Health <- cut(df$Self_Assessed_Health, breaks=c(1,3,6),

right=FALSE, labels=c('Good', 'Moderate or Poor'))

df$Trust_Health_Care <- cut(df$Trust_Health_Care, breaks=c(1,3,6), right=FALSE,

labels=c('A lot of trust', 'Little trust'))

df$Concern_Covid <- cut(df$Concern_Covid, breaks=c(1,3,6), right=FALSE,

labels=c('A little or not', 'Very'))

df$Seriousness_Covid <- as.numeric(df$Seriousness_Covid)

df$Seriousness_Covid <- cut(df$Seriousness_Covid, breaks=c(1,3,4,6), right=FALSE,

labels = c('Serious','Neutral', 'Exaggerated'))

df$Gender2[df$Gender == 1] <- 'Male'

df$Gender2[df$Gender == 2] <- 'Female'

df$Gender <- as.factor(df$Gender2)

# Recode distance so 1 is positve and 2 is negative

df$c_distance_ic2[df$c_distance_ic == 2] <- 1

df$c_distance_ic2[df$c_distance_ic==1] <- 2

df$c_distance_ic<-as.factor(df$c_distance_ic2)

df$c_distance_vac2[df$c_distance_vac == 2] <- 1

df$c_distance_vac2[df$c_distance_vac==1] <- 2

df$c_distance_vac<-as.factor(df$c_distance_vac2)

# Make list to add labels to the plots

levels.ic<-list()

levels.ic[["a_age_ic"]]<-c("27 years old","52 years old",'77 years old')

levels.ic[['b_weight_ic']]<-c("Healthy weight (BMI = 22)",

"Overweight (BMI = 31)")

levels.ic[["c_distance_ic"]]<-c("Obeyed the measures","Disobeyed the measures")

levels.ic[['d_occupation_ic']]<-c('Nurse', 'Teacher', 'Administrative assistant',

'Unemployed')

levels.vac<-list()

levels.vac[["a_age_vac"]]<-c("27 years old","52 years old",'77 years old')

levels.vac[['b_weight_vac']]<-c("Healthy weight (BMI = 22)",

"Overweight (BMI = 31)")

levels.vac[["c_distance_vac"]]<-c("Obeyed the measures","Disobeyed the measures")

levels.vac[['d_occupation_vac']]<-c('Nurse', 'Teacher', 'Administrative assistant',

'Unemployed')

## AVERAGE MARGINAL CONDITION EFFECT INTENSIVE CARE ---------------------------

# AMCE unconditional IC

results_ic <- amce(outcome_ic ~ a_age_ic + b_weight_ic + c_distance_ic + d_occupation_ic,

data = df, respondent.id= 'ID', na.ignore = TRUE, weights = 'data_weights')

# Print summary

summary(results_ic)

plot(results_ic, xlab="Effect on Pr(Preffered for IC)",

attribute.names = c('Age','Weight', 'Covid-19 Measures', 'Occupation'),

level.names = levels.ic, xlim=c(-.5,.5), breaks=c(-.4, 0, .4),

labels=c("-.4","0",".4"), point.size = 0.4, text.size = 12,

label.baseline = FALSE, col = 'black', ci=.95)

## Interactions Age

ic_interaction_age <- amce(outcome_ic ~ a_age_ic + b_weight_ic + c_distance_ic +

d_occupation_ic + a_age_ic*Generation +

b_weight_ic*Generation + c_distance_ic*Generation +

d_occupation_ic*Generation, data=subset(df, !is.na(df$Generation)),

respondent.id="ID", na.ignore=TRUE,

respondent.varying=c("Generation"), cluster = FALSE)

summary(ic_interaction_age)

# Plot results

plot(ic_interaction_age, xlab="Effect on Pr(Preffered for IC)",

attribute.names = c('Age','Weight', 'Covid-19 Measures', 'Occupation'),

level.names = levels.ic, xlim=c(-.5,.5), breaks=c(-.4, 0, .4),

labels=c("-.4","0",".4"), point.size = 0.4, text.size = 12,

label.baseline = FALSE,col = 'black', plot.display = 'interaction',

ci=.95)

#-------------------------------------------------------------------------------

## Interactions Distance Respondent

ic_interaction_Obeying_Distance <-amce(outcome_ic ~ a_age_ic + b_weight_ic +

c_distance_ic + d_occupation_ic +

a_age_ic*Obeying_Distance + b_weight_ic*Obeying_Distance +

c_distance_ic*Obeying_Distance +

d_occupation_ic*Obeying_Distance,

data=subset(df,!is.na(df$Obeying_Distance)),

respondent.id="ID", na.ignore=TRUE,

respondent.varying=c("Obeying_Distance"),

cluster = FALSE)

summary(ic_interaction_Obeying_Distance)

# Plot results

plot(ic_interaction_Obeying_Distance, xlab="Effect on Pr(Preffered for IC)",

attribute.names = c('Age','Weight', 'Covid-19 Measures', 'Occupation'),

level.names = levels.ic, xlim=c(-.5,.5), breaks=c(-.4, 0, .4),

labels=c("-.4","0",".4"), point.size = 0.4, text.size = 12,

label.baseline = FALSE,col = 'black', plot.display = 'interaction',

ci=.95)

#-------------------------------------------------------------------------------

## Interactions Political Preference

ic_interaction_Political_Preference <-amce(outcome_ic ~ a_age_ic + b_weight_ic +

c_distance_ic + d_occupation_ic +

a_age_ic*Political_Preference +

b_weight_ic*Political_Preference +

c_distance_ic*Political_Preference +

d_occupation_ic*Political_Preference,

data=subset(df, !is.na(df$Political_Preference)),

respondent.id="ID", na.ignore=TRUE,

respondent.varying=c("Political_Preference"),

cluster=FALSE)

summary(ic_interaction_Political_Preference)

# Plot results

plot(ic_interaction_Political_Preference, xlab="Effect on Pr(Preffered for IC)",

attribute.names = c('Age','Weight', 'Covid-19 Measures', 'Occupation'),

level.names = levels.ic, xlim=c(-.5,.5), breaks=c(-.4, 0, .4),

labels=c("-.4","0",".4"), point.size = 0.4, text.size = 12,

label.baseline = FALSE,col = 'black', plot.display = 'interaction',

ci=.95)

#-------------------------------------------------------------------------------

## Interactions Educational Level Respondent

ic_interaction_Educational_Attainment <-amce(outcome_ic ~ a_age_ic + b_weight_ic +

c_distance_ic +

d_occupation_ic + a_age_ic*Educational_Attainment +

b_weight_ic*Educational_Attainment +

c_distance_ic*Educational_Attainment +

d_occupation_ic*Educational_Attainment,

data=subset(df,!is.na(df$Educational_Attainment)),

respondent.id="ID", na.ignore=TRUE,

respondent.varying=c("Educational_Attainment"),

cluster = FALSE)

summary(ic_interaction_Educational_Attainment)

# Plot results

plot(ic_interaction_Educational_Attainment, xlab="Effect on Pr(Preffered for IC)",

attribute.names = c('Age','Weight', 'Covid-19 Measures', 'Occupation'),

level.names = levels.ic, xlim=c(-.5,.5), breaks=c(-.4, 0, .4),

labels=c("-.4","0",".4"), point.size = 0.4, text.size = 12,

label.baseline = FALSE,col = 'black', plot.display = 'interaction',

ci=.95)

#-------------------------------------------------------------------------------

## Interactions Health Respondent

ic_interaction_Self_Assessed_Health <-amce(outcome_ic ~ a_age_ic + b_weight_ic +

c_distance_ic +

d_occupation_ic + a_age_ic*Self_Assessed_Health +

b_weight_ic*Self_Assessed_Health +

c_distance_ic*Self_Assessed_Health +

d_occupation_ic*Self_Assessed_Health,

data=subset(df,!is.na(df$Self_Assessed_Health)),

respondent.id="ID",na.ignore=TRUE,

respondent.varying=c("Self_Assessed_Health"),

cluster = FALSE)

summary(ic_interaction_Self_Assessed_Health)

# Plot results

plot(ic_interaction_Self_Assessed_Health, xlab="Effect on Pr(Preffered for IC)",

attribute.names = c('Age','Weight', 'Covid-19 Measures', 'Occupation'),

level.names = levels.ic, xlim=c(-.5,.5), breaks=c(-.4, 0, .4),

labels=c("-.4","0",".4"), point.size = 0.4, text.size = 12,

label.baseline = FALSE,col = 'black', plot.display = 'interaction',

ci=.95)

#-------------------------------------------------------------------------------

# Interactions Trusth Health Care Respondent

ic_interaction_Trust_Health_Care <-amce(outcome_ic ~ a_age_ic + b_weight_ic +

c_distance_ic + d_occupation_ic +

a_age_ic*Trust_Health_Care +

b_weight_ic*Trust_Health_Care +

c_distance_ic*Trust_Health_Care +

d_occupation_ic*Trust_Health_Care,

data=subset(df,!is.na(df$Trust_Health_Care)),

respondent.id="ID",na.ignore=TRUE,

respondent.varying=c("Trust_Health_Care"),

cluster = FALSE)

summary(ic_interaction_Trust_Health_Care)

# Plot results

plot(ic_interaction_Trust_Health_Care, xlab="Effect on Pr(Preffered for IC)",

attribute.names = c('Age','Weight', 'Covid-19 Measures', 'Occupation'),

level.names = levels.ic, xlim=c(-.5,.5), breaks=c(-.4, 0, .4),

labels=c("-.4","0",".4"), point.size = 0.4, text.size = 12,

label.baseline = FALSE,col = 'black', plot.display = 'interaction',

ci=.95)

#-------------------------------------------------------------------------------

# Interactions Gender Respondent

ic_interaction_gender <-amce(outcome_ic ~ a_age_ic + b_weight_ic + c_distance_ic +

d_occupation_ic + a_age_ic*Gender +

b_weight_ic*Gender + c_distance_ic*Gender +

d_occupation_ic*Gender,

data=subset(df,!is.na(df$Gender)),

respondent.id="ID",na.ignore=TRUE,

respondent.varying=c("Gender"),

cluster = FALSE)

summary(ic_interaction_gender)

# Plot results

plot(ic_interaction_gender, xlab="Effect on Pr(Preffered for IC)",

attribute.names = c('Age','Weight', 'Covid-19 Measures', 'Occupation'),

level.names = levels.ic, xlim=c(-.5,.5), breaks=c(-.4, 0, .4),

labels=c("-.4","0",".4"), point.size = 0.4, text.size = 12,

label.baseline = FALSE,col = 'black', plot.display = 'interaction',

ci=.95)

#-------------------------------------------------------------------------------

# Interactions Concern COVID

ic_interaction_Concern_Covid <-amce(outcome_ic ~ a_age_ic + b_weight_ic + c_distance_ic +

d_occupation_ic + a_age_ic*Concern_Covid +

b_weight_ic*Concern_Covid + c_distance_ic*Concern_Covid +

d_occupation_ic*Concern_Covid,

data=subset(df,!is.na(df$Concern_Covid)),

respondent.id="ID",na.ignore=TRUE,

respondent.varying=c("Concern_Covid"),

cluster = FALSE)

summary(ic_interaction_Concern_Covid)

# Plot results

plot(ic_interaction_Concern_Covid, xlab="Effect on Pr(Preffered for IC)",

attribute.names = c('Age','Weight', 'Covid-19 Measures', 'Occupation'),

level.names = levels.ic, xlim=c(-.5,.5), breaks=c(-.4, 0, .4),

labels=c("-.4","0",".4"), point.size = 0.4, text.size = 12,

label.baseline = FALSE,col = 'black', plot.display = 'interaction',

ci=.95)

#-------------------------------------------------------------------------------

# Interactions opinion COVID

ic_interaction_Seriousness_Covid <-amce(outcome_ic ~ a_age_ic + b_weight_ic +

c_distance_ic + d_occupation_ic +

a_age_ic*Seriousness_Covid +

b_weight_ic*Seriousness_Covid +

c_distance_ic*Seriousness_Covid +

d_occupation_ic*Seriousness_Covid,

data=subset(df,!is.na(df$Seriousness_Covid)),

respondent.id="ID",na.ignore=TRUE,

respondent.varying=c("Seriousness_Covid"),

cluster = FALSE)

summary(ic_interaction_Seriousness_Covid)

# Plot results

plot(ic_interaction_Seriousness_Covid, xlab="Effect on Pr(Preffered for IC)",

attribute.names = c('Age','Weight', 'Covid-19 Measures', 'Occupation'),

level.names = levels.ic, xlim=c(-.5,.5), breaks=c(-.4, 0, .4),

labels=c("-.4","0",".4"), point.size = 0.4, text.size = 12,

label.baseline = FALSE,col = 'black', plot.display = 'interaction',

ci=.95)

#-------------------------------------------------------------------------------

## AVERAGE MARGINAL CONDITION EFFECT VACCIN ---------------------------------

# AMCE Unconditional Vaccin

results_vac <- amce(outcome_vac ~ a_age_vac + b_weight_vac + c_distance_vac + d_occupation_vac,

data = df, respondent.id= 'ID', na.ignore = TRUE, weights='data_weights')

summary(results_vac)

# Plot results

plot(results_vac, xlab="Effect on Pr(Preffered for vaccine)",

attribute.names = c('Age','Weight', 'Covid-19 Measures', 'Occupation'),

level.names = levels.vac, xlim=c(-.5,.5), breaks=c(-.4, 0, .4),

labels=c("-.4","0",".4"), point.size = 0.4, text.size = 12,

label.baseline = FALSE,col = 'black', ci=.95)

#--------------------------------------------------------------------------------

## Interactions Age

vac_interaction_age <- amce(outcome_vac ~ a_age_vac + b_weight_vac + c_distance_vac +

d_occupation_vac + a_age_vac*Generation +

b_weight_vac*Generation + c_distance_vac*Generation +

d_occupation_vac*Generation,

data=subset(df,!is.na(df$Generation)), respondent.id="ID",

na.ignore=TRUE, respondent.varying=c("Generation"),

cluster = FALSE)

summary(vac_interaction_age)

# Plot results

plot(vac_interaction_age, xlab="Effect on Pr(Preffered for vaccine)",

attribute.names = c('Age','Weight', 'Covid-19 Measures', 'Occupation'),

level.names = levels.vac, xlim=c(-.5,.5), breaks=c(-.4, 0, .4),

labels=c("-.4","0",".4"), point.size = 0.4, text.size = 12,

label.baseline = FALSE,col = 'black', plot.display = 'interaction',

ci=.95)

#-------------------------------------------------------------------------------

## Interactions Distance Respondent

vac_interaction_Obeying_Distance <-amce(outcome_vac ~ a_age_vac + b_weight_vac +

c_distance_vac +

d_occupation_vac + a_age_vac*Obeying_Distance +

b_weight_vac*Obeying_Distance +

c_distance_vac*Obeying_Distance +

d_occupation_vac*Obeying_Distance,

data=subset(df,!is.na(df$Obeying_Distance)),

respondent.id="ID", na.ignore=TRUE,

respondent.varying=c("Obeying_Distance"),

cluster = FALSE)

summary(vac_interaction_Obeying_Distance)

# Plot results

plot(vac_interaction_Obeying_Distance, xlab="Effect on Pr(Preffered for vaccine)",

attribute.names = c('Age','Weight', 'Covid-19 Measures', 'Occupation'),

level.names = levels.vac, xlim=c(-.5,.5), breaks=c(-.4, 0, .4),

labels=c("-.4","0",".4"), point.size = 0.4, text.size = 12,

label.baseline = FALSE,col = 'black', plot.display = 'interaction',

ci=.95)

#-------------------------------------------------------------------------------

## Interactions Political Preference

vac_interaction_Political_Preference <-amce(outcome_vac ~ a_age_vac + b_weight_vac +

c_distance_vac +

d_occupation_vac + a_age_vac*Political_Preference +

b_weight_vac*Political_Preference +

c_distance_vac*Political_Preference +

d_occupation_vac*Political_Preference,

data=subset(df,!is.na(df$Political_Preference)),

respondent.id="ID",

na.ignore=TRUE, respondent.varying=c("Political_Preference"),

cluster=FALSE)

summary(vac_interaction_Political_Preference)

# Plot results

plot(vac_interaction_Political_Preference, xlab="Effect on Pr(Preffered for vaccine)",

attribute.names = c('Age','Weight', 'Covid-19 Measures', 'Occupation'),

level.names = levels.vac, xlim=c(-.5,.5), breaks=c(-.4, 0, .4),

labels=c("-.4","0",".4"), point.size = 0.4, text.size = 12,

label.baseline = FALSE,col = 'black', plot.display = 'interaction',

ci=.95)

#-------------------------------------------------------------------------------

## Interactions Educational Level Respondent

vac_interaction_Educational_Attainment <-amce(outcome_vac ~ a_age_vac + b_weight_vac +

c_distance_vac + d_occupation_vac +

a_age_vac*Educational_Attainment +

b_weight_vac*Educational_Attainment +

c_distance_vac*Educational_Attainment +

d_occupation_vac*Educational_Attainment,

data=subset(df, !is.na(df$Educational_Attainment)),

respondent.id="ID", na.ignore=TRUE,

respondent.varying=c("Educational_Attainment"))

summary(vac_interaction_Educational_Attainment)

# Plot results

plot(vac_interaction_Educational_Attainment, xlab="Effect on Pr(Preffered for vaccine)",

attribute.names = c('Age','Weight', 'Covid-19 Measures', 'Occupation'),

level.names = levels.vac, xlim=c(-.5,.5), breaks=c(-.4, 0, .4),

labels=c("-.4","0",".4"), point.size = 0.4, text.size = 12,

label.baseline = FALSE,col = 'black', plot.display = 'interaction',

ci=.95)

#-------------------------------------------------------------------------------

## Interactions Health Respondent

vac_interaction_health <- amce(outcome_vac ~ a_age_vac + b_weight_vac + c_distance_vac +

d_occupation_vac + a_age_vac*Self_Assessed_Health +

b_weight_vac*Self_Assessed_Health +

c_distance_vac*Self_Assessed_Health +

d_occupation_vac*Self_Assessed_Health,

data=subset(df, !is.na(df$Self_Assessed_Health)),

respondent.id="ID",na.ignore=TRUE,

respondent.varying=c("Self_Assessed_Health"),

cluster = FALSE)

summary(vac_interaction_health)

# Plot results

plot(vac_interaction_health, xlab="Effect on Pr(Preffered for vaccine)",

attribute.names = c('Age','Weight', 'Covid-19 Measures', 'Occupation'),

level.names = levels.vac, xlim=c(-.5,.5), breaks=c(-.4, 0, .4),

labels=c("-.4","0",".4"), point.size = 0.4, text.size = 12,

label.baseline = FALSE,col = 'black', plot.display = 'interaction',

ci=.95)

#-------------------------------------------------------------------------------

# Interactions Trusth Health Care Respondent

vac_interaction_Trust_Health_Care <-amce(outcome_vac ~ a_age_vac + b_weight_vac +

c_distance_vac + d_occupation_vac +

a_age_vac*Trust_Health_Care +

b_weight_vac*Trust_Health_Care +

c_distance_vac*Trust_Health_Care +

d_occupation_vac*Trust_Health_Care,

data=subset(df,!is.na(df$Trust_Health_Care)),

respondent.id="ID",na.ignore=TRUE,

respondent.varying=c("Trust_Health_Care"),

cluster = FALSE)

summary(vac_interaction_Trust_Health_Care)

# Plot results

plot(vac_interaction_Trust_Health_Care, xlab="Effect on Pr(Preffered for vaccine)",

attribute.names = c('Age','Weight', 'Covid-19 Measures', 'Occupation'),

level.names = levels.vac, xlim=c(-.5,.5), breaks=c(-.4, 0, .4),

labels=c("-.4","0",".4"), point.size = 0.4, text.size = 12,

label.baseline = FALSE,col = 'black', plot.display = 'interaction',

ci=.95)

#-------------------------------------------------------------------------------

# Interactions Gender Respondent

vac_interaction_gender <-amce(outcome_vac ~ a_age_vac + b_weight_vac + c_distance_vac +

d_occupation_vac + a_age_vac*Gender +

b_weight_vac*Gender + c_distance_vac*Gender +

d_occupation_vac*Gender,

data=subset(df,!is.na(df$Gender)),

respondent.id="ID",na.ignore=TRUE,

respondent.varying=c("Gender"),

cluster = FALSE)

summary(vac_interaction_gender)

# Plot results

plot(vac_interaction_gender, xlab="Effect on Pr(Preffered for vaccine)",

attribute.names = c('Age','Weight', 'Covid-19 Measures', 'Occupation'),

level.names = levels.vac, xlim=c(-.5,.5), breaks=c(-.4, 0, .4),

labels=c("-.4","0",".4"), point.size = 0.4, text.size = 12,

label.baseline = FALSE,col = 'black', plot.display = 'interaction',

ci=.95)

#-------------------------------------------------------------------------------

# Interactions Concern COVID

vac_interaction_Concern_Covid <-amce(outcome_vac ~ a_age_vac + b_weight_vac +

c_distance_vac + d_occupation_vac +

a_age_vac*Concern_Covid +

b_weight_vac*Concern_Covid +

c_distance_vac*Concern_Covid +

d_occupation_vac*Concern_Covid,

data=subset(df,!is.na(df$Concern_Covid)),

respondent.id="ID",na.ignore=TRUE,

respondent.varying=c("Concern_Covid"),

cluster = FALSE)

summary(vac_interaction_Concern_Covid)

# Plot results

plot(vac_interaction_Concern_Covid,xlab="Effect on Pr(Preffered for vaccine)",

attribute.names = c('Age','Weight', 'Covid-19 Measures', 'Occupation'),

level.names = levels.vac, xlim=c(-.5,.5), breaks=c(-.4, 0, .4),

labels=c("-.4","0",".4"), point.size = 0.4, text.size = 12,

label.baseline = FALSE,col = 'black', plot.display = 'interaction',

ci=.95)

#-------------------------------------------------------------------------------

# Interactions opinion COVID

vac_interaction_Seriousness_Covid <-amce(outcome_vac ~ a_age_vac + b_weight_vac +

c_distance_vac + d_occupation_vac +

a_age_vac*Seriousness_Covid +

b_weight_vac*Seriousness_Covid +

c_distance_vac*Seriousness_Covid +

d_occupation_vac*Seriousness_Covid,

data=subset(df,!is.na(df$Seriousness_Covid)),

respondent.id="ID",na.ignore=TRUE,

respondent.varying=c("Seriousness_Covid"),

cluster = FALSE)

summary(vac_interaction_Seriousness_Covid)

# Plot results

plot(vac_interaction_Seriousness_Covid,xlab="Effect on Pr(Preffered for vaccine)",

attribute.names = c('Age','Weight', 'Covid-19 Measures', 'Occupation'),

level.names = levels.vac, xlim=c(-.5,.5), breaks=c(-.4, 0, .4),

labels=c("-.4","0",".4"), point.size = 0.4, text.size = 12,

label.baseline = FALSE,col = 'black', plot.display = 'interaction',

ci=.95)
